# Polyploid QTL-seq identified QTLs controlling potato flesh color and tuber starch phosphorus content in a plexity-dependent manner

**DOI:** 10.1270/jsbbs.24028

**Published:** 2024-11-23

**Authors:** Hiromoto Yamakawa, Tatsumi Mizubayashi, Noriyuki Kitazawa, Utako Yamanouchi, Tsuyu Ando, Yoshiyuki Mukai, Etsuo Shimosaka, Takahiro Noda, Kenji Asano, Kotaro Akai, Kenji Katayama

**Affiliations:** 1 Institute of Crop Science, National Agriculture and Food Research Organization (NARO), 2-1-2 Kannondai, Tsukuba, Ibaraki 305-8518, Japan; 2 Hokkaido Agricultural Research Center, National Agriculture and Food Research Organization (NARO), 9-4 Shinseiminami, Memuro, Kasai, Hokkaido 082-0081, Japan

**Keywords:** polyploid, QTL-seq, DNA marker, potato, flesh color, starch phosphorus content, plexity

## Abstract

The progenies of polyploid crops inherit multiple sets of homoeologous chromosomes through various combinations, which impedes the identification of the quantitative trait loci (QTL) governing agronomic traits and the implementation of DNA marker-assisted breeding. Previously, we developed a whole-genome sequencing-based polyploid QTL-seq method that utilizes comprehensively extracted simplex polymorphisms for QTL mapping. Here, we verified the detection of duplex QTLs by modifying the analytical settings to explore the QTLs governing tuber flesh color and starch phosphorus content using tetraploid potato (*Solanum tuberosum* L.). The F_1_ progenies were obtained from a cross between ‘Touya’ (TY) and ‘Benimaru’ (BM). A single TY-derived QTL responsible for yellow flesh color was identified around a β-carotene hydroxylase gene on chromosome 3 using simplex polymorphisms, and a BM-derived QTL associated with decreased starch phosphorus content near a starch synthase II gene on chromosome 2 was detected using duplex polymorphisms. Furthermore, linked DNA markers were developed at the QTL sites. For the latter QTL, plexity-distinguishable markers were developed using quantitative PCR, fragment analysis, and amplicon sequencing. These revealed the allele dosage-dependent effect of the reduced starch phosphorus content. Thus, the polyploid QTL-seq pipeline can explore versatile QTLs beyond simplex, facilitating DNA marker-assisted breeding in various polyploid crops.

## Introduction

Potato (*Solanum tuberosum* L.) is an important tuberous crop, with a global production of approximately 370 million tons across Asian, European, and American countries (FAOSTAT 2022). It is a staple food in various cuisines and widely used in processed products, including potato chips and French fries. In Japan, different potato varieties are available with diverse flesh colors, including white, yellow, red, and purple. The yellow pigments in cultivated tetraploid potato consist of carotenoids, such as lutein and violaxanthin, while the orange-colored tubers of the diploid progenies of *S. phureja* contain zeaxanthin (Kobayashi *et al.* 2008, Morris *et al.* 2004, Nesterenko and Sink 2003). The desired flesh color, a crucial breeding trait, depends on the usage of the cultivars. Furthermore, lutein and zeaxanthin have health benefits, such as improving vision. Additionally, potatoes are a vital starch source, containing significant amounts of covalently bound phosphate. Phosphate in starch enhances the water-binding capacity, freeze–thaw stability, and viscosity of its gel (Ramadan and Sitohy 2020), making it suitable for various applications, such as making fish paste and noodles. Starch with low phosphate content is preferred for frozen fish paste products due to less syneresis during the freeze–thawing process, while that with high phosphate content enhances the firmness of instant noodles (Noda *et al.* 2006b). Thus, the starch phosphate content determines the quality of potato starch.

Ongoing breeding efforts aim to improve potato quality, including flesh color and starch characteristics. To expedite the breeding processes, the QTLs associated with agronomically important traits should be explored and incorporated into elite cultivars through DNA marker-assisted selection. However, the polyploid nature of cultivated potatoes, which possess four sets of homoeologous chromosomes with similar DNA sequences, complicates genetic dissection using DNA markers.

Next-generation sequencing has revolutionized genetic analysis in crops, including polyploid ones like potato. The reference genome sequence established for doubled monoploid potato *S. tuberosum* Group *Phureja* DM 1-3 516 R44 (Pham *et al.* 2020, Potato Genome Sequencing Consortium 2011) has facilitated genetic mapping of traits through DNA sequence verification. Several tools, such as QTLpoly (Pereira *et al.* 2020) and GWASpoly (Rosyara *et al.* 2016), have been developed to identify genomic regions related to important traits in polyploid crops. The QTLs for flesh color, tuber shape, and disease resistance have been successfully identified in potato (Aalborg *et al.* 2024, Koizumi *et al.* 2021, Pandey *et al.* 2022, Park *et al.* 2021, 2023, 2024, Rosyara *et al.* 2016). Recently, we developed a whole-genome sequencing-based bulked segregant analysis method called polyploid QTL-seq, enabling rapid identification of QTLs in polyploid crops (Yamakawa *et al.* 2021). It is an extension of the original QTL-seq program (Sugihara *et al.* 2022, Takagi *et al.* 2013) tailored for polyploid F_1_ populations. The program pipeline streamlines the analysis with a few commands (Yamakawa *et al.* 2024) and includes a plexity-adapted simulation based on an F_1_ null distribution, assuming the absence of QTLs, to compute *P* values. The program generates SNP- and ΔSNP-index plots and a list of QTL-deduced variants. Polyploid QTL-seq, which utilizes unique simplex single nucleotide polymorphisms (SNPs) exclusively located on a single homoeologous chromosome for genetic trait dissection, has been successfully used to identify QTLs for flesh color and steamed tuber texture in hexaploid sweetpotato (Yamakawa *et al.* 2021, 2024). However, previous analyses might have overlooked some QTLs with multiplex genotypes.

Current breeding programs for disease-resistant potato cultivars routinely utilize DNA markers to generate resistance against the potato cyst nematode, late blight, and potato viruses X and Y (Asano *et al.* 2012, 2021, Asano and Tamiya 2016, Mori *et al.* 2011, 2015). As resistance genes are gathered with multiplex genotypes to increase the frequency of resistant progenies and establish preferable parental lines, breeding programs need to handle multiplexed alleles. Additionally, polyploidy is observed in fruit crops, such as strawberry and persimmon. During the breeding of hexaploid persimmon, a dominant astringency trait is removed using repetitive inbreeding to obtain null segregants as nonastringent cultivars. However, this can suppress yield-related traits (Onoue *et al.* 2022), necessitating the exploration of yield-enhancing QTLs. In such cases, analysis methods dealing with multiplexed alleles accumulated through repetitive inbreeding are required.

This study aimed to verify the detection of QTLs with multiplex genotypes in potato using polyploid QTL-seq and to develop DNA markers to distinguish the plexities of these QTLs. Application of this method to potato progenies derived from a cross between tuber parents with different flesh color and starch phosphorus content demonstrated that these traits were regulated by a single simplex QTL and a duplex QTL, respectively. Furthermore, DNA markers distinguishing the plexity of the latter QTL were developed using several methods, such as quantitative PCR, fragment analysis, and amplicon sequencing, and the results were compared.

## Materials and Methods

### Plant materials

The 178 F_1_ progenies resulting from crosses between *S. tuberosum* cv. ‘Benimaru’ (BM; white flesh and low starch phosphorus content) and cv. ‘Touya’ (TY; yellow flesh and high starch phosphorus content) were cultivated in the field. The tubers were harvested and stored at 4°C for approximately one week before flesh color inspection and starch preparation. The mean values of the phenotypic measurements conducted in 2022 and 2023 were analyzed as follows.

### Evaluation of tuber flesh color

After vertically cutting the tubers in halves, the section surfaces were photographed. Then, the International Commission on Illumination CIE 1976 color space values (L*, a*, and b*) were measured on the perimedullary zone between the pith and cortex using a CR-20 color reader (Konica Minolta, Inc., Tokyo, Japan). The results were described as L* (brightness or lightness, positive toward white and negative toward black), a* (red–green, positive toward red and negative toward green), b* (yellow–blue, positive toward yellow and negative toward blue), and C* [chroma, calculated as the square root value of (a*)^2^ + (b*)^2^] (McGuire 1992).

### Preparation of tuber starch and measurement of phosphorus content

For starch preparation, two tubers (approximately 150 g) were washed, diced into small pieces, and homogenized with 200 mL of distilled water in a mixer (SKS-H700; TIGER, Osaka, Japan). The resultant slurry was filtered through a 150 μm metallic sieve to allow most starch granules to pass. After allowing the starch suspension to stand for 2 h, the starch granules were recovered by decantation. The residual starch pellets were washed thrice with distilled water and dried under vacuum. Purified starch samples were stored at 4°C for subsequent analysis.

The starch phosphorus content was determined using energy-dispersive X-ray fluorescence spectroscopy as described previously (Noda *et al.* 2006a). Briefly, each starch sample (approximately 4 g) was converted into pellets (22 mm in diameter) using a J-15 high-pressure jack (AS ONE Corporation, Osaka, Japan). The phosphorus content was quantitatively analyzed at an accelerating voltage of 15 kV and a detection time of 200 s with a spectrometer (EDX-700; Shimadzu, Kyoto, Japan). The number of P Kα counts per second (cps/μA) was detected.

The absolute phosphorus content of the BM and TY starch samples was determined using inductively coupled plasma-optical emission spectrometry. The starch samples (2 g) were ashed at 500°C for 10 h and evaporated with hydrochloric acid. After extracting phosphorus using hydrochloric acid from the residue and filtering it, the phosphorus content was determined at an RF power of 1,100 W, a plasma flow of 15 L/min, an auxiliary flow of 1.2 L/min, a nebulizer flow of 0.8 L/min, a plasma view of axial mode, and a detection wavelength of 213.62 nm with a spectrometer (ICPE-9820; Shimadzu, Kyoto, Japan).

### Genomic DNA extraction, bulking, and sequencing

Genomic DNA was extracted from the unexpanded leaves of the shoot apex of the F_1_ progenies and their parents, employing a DNeasy Plant Mini Kit (QIAGEN, Hilden, Germany). Bulk DNA samples were prepared by combining equivalent quantities of DNA from the F_1_ individuals. Sequence libraries were obtained using a TruSeq DNA PCR-Free Sample Preparation Kit (Illumina, San Diego, CA, USA) and subjected to 150-bp paired-end sequencing using an Illumina NovaSeq6000 DNA sequencer.

### Polyploid QTL-seq analysis

Polyploid QTL-seq analysis, developed from an original diploid-restricted QTL-seq (Sugihara *et al.* 2022, Takagi *et al.* 2013) by implementing plexity-selecting filters, was executed using the polyploid QTL-seq pipeline ver 1.0.0, available on GitHub (https://github.com/TatsumiMizubayashi/PolyploidQtlSeq). The analysis was performed on an iMac Pro computer with a 36-thread Intel Xeon W processor, 128 GB RAM, and 4 TB HDD. The pipeline integrates the following programs: .NET6 version 6.0.400, fastp version 0.23.2, bwa version 0.7.17-r1188, samtools version 1.15.1, bcftools version 1.15.1, bgzip, tabix, and snpEff version 5.0. The read quality for parents and all bulks was assessed using default settings. Whereas for the QTL-seq analysis with simplex variants, the configuration parameters were modified as follows: minMQ = 40, adjustMQ = 60, p2SnpIndexRange = 0.10–0.36, minDepth = 40, and ploidy = 4. In the case of analyses involving duplex variants, the p2SnpIndexRange parameter was adjusted to 0.37–0.63, while the NPlex parameter was at 2. The chromosome-scale genome sequence of the doubled monoploid potato *S. tuberosum* Group Phureja DM 1-3 516 R44 v6.1 (Pham *et al.* 2020) was used as a reference. The analysis was repeated with the P1 and P2 parents swapped to explore QTLs originating from both parents. Supplemental Table 1 provides the variant counts employed in each QTL analysis. QTL candidate regions were delineated based on the criterion that the P99 + or – ΔSNP-index QTL variant count, that is the number of QTL-deduced variants with positive or negative ΔSNP-index by the P99 criteria in the 100-kb sliding window (as listed in the SlidingWindow.txt file generated by the polyploid QTL-seq pipeline) exceeded 100 in one direction of QTL effect but not in the other. A comprehensive protocol is available in the user manual on GitHub (https://github.com/TatsumiMizubayashi/PolyploidQtlSeq).

### Preparation of linked DNA markers and genotyping of F_1_ individuals

PCR primers for the SNP sites in the middle of the QTL-seq-identified candidate regions (Supplemental Table 2) were used to assess the effects of the QTLs on the traits.

For the DNA marker corresponding to the simplex QTL associated with flesh color, PCR was performed using a mixture (total 10 μL) of 1× GoTaq Hot Start Colorless Master Mix (Promega, Madison, WI, USA), 10 ng of genomic DNA from each F_1_ individual, and 0.2 μM each of forward and reverse primers (or those for *GBSS* as a control). The cycling parameters were as follows: initial denaturation at 95°C for 2 min; 30 cycles consisting of denaturation at 95°C for 30 s, annealing at 55°C for 30 s, and extension at 72°C for 30 s (1 min for *GBSS*); and a final extension at 72°C for 2 min. The resultant amplicons were verified by electrophoresis on a 1.5% agarose gel.

For analyzing the duplex QTLs corresponding to starch phosphorus content, quantitative PCR was performed using a C1000 Touch Thermal Cycler/CFX384 Real-Time System (BIO-RAD, Hercules, CA, USA) with THUNDERBIRD SYBR qPCR Mix (TOYOBO, Osaka, Japan) and the alternative base (ALT)-specific primer set. PCR mixtures (total 12 μL) comprised 1× THUNDERBIRD SYBR qPCR Mix, 10 ng of genomic DNA from each F_1_ individual, and 0.2 μM each of forward and reverse primers. The cycling parameter was as follows: 95°C for 60 s and 40 cycles of 95°C for 15 s, 55°C for 30 s, and 72°C for 45 s. The allele dosage was calculated using the method by Pfaffl (2001).

Fragment analysis in which the amplicon with the alternative base (ALT) gave a 3-base larger fragment than that with the reference base (REF) was conducted in a 10-μL mixture containing 1× GoTaq Hot Start Colorless Master Mix (Promega), 10 ng of genomic DNA from each F_1_ individual, 50 nM each of forward primers with M13 tail at the 5ʹ-end and a reverse primer, and 25 nM 6-FAM-labeled universal M13 primer. The cycling conditions were as follows: initial denaturation at 95°C for 2 min; 30 cycles of 95°C for 30 s, 55°C for 30 s, and 72°C for 30 s; 3 cycles of 95°C for 30 s, 50°C for 30 s, and 72°C for 30 s; and a final extension at 72°C for 2 min. The DNA amplicons were separated and detected using an Applied Biosystems 3730xl DNA Analyzer (Thermo Fisher Scientific Inc., Waltham, MA, USA) with a 36-cm capillary array and POP-7 polymer. The size of each amplified band was estimated based on a set of internal standard DNA fragments (GeneScan 400HD ROX Dye Size Standard; Thermo Fisher Scientific Inc.). Data were analyzed using GeneMapper v. 5.0 software. The ratio of the height of the alternative base (ALT) peak to that of the reference base (REF) peak was calculated.

### Genotyping of F_1_ individuals by amplicon sequencing

Amplicon sequencing was conducted employing a two-step tailed PCR approach. The initial PCR used forward and reverse primers appended with Nextera R1 and R2 tags, respectively, facilitating subsequent adapter addition during the second round of PCR according to the protocol of Nextera XT Index Kit v2 (Illumina). The first PCR reaction mixture (total volume of 10 μL) comprised 1× GoTaq Hot Start Colorless Master Mix (Promega), 10 ng of genomic DNA from each F_1_ individual, and 0.2 μM each of the forward and reverse primers. The first PCR program was as follows: initial denaturation at 95°C for 2 min, followed by 30 cycles of 95°C for 30 s, 55°C for 30 s, and 72°C for 30 s, and a final extension at 72°C for 2 min. Three distinct target regions (Chr02:41322042, Chr02:41426723, and Chr02:41523385) were amplified independently. Then, the resultant products were diluted 100-fold with sterilized distilled water combined, and 2 μL of the diluted product was used as the template for the second PCR. To perform bidirectional amplicon tagging in the second PCR, a forward fusion primer incorporating the Illumina P5 adapter, barcode, and the Nextera R1 tag and a reverse fusion primer containing the Illumina P7 adapter, barcode, and the Nextera R2 tag were utilized. The 10-μL second PCR mixture consisted of 1× GoTaq Hot Start Colorless Master Mix (Promega) and 0.5 μM each of the forward and reverse fusion primers. The PCR conditions were as follows: 95°C for 2 min, followed by 15 cycles of 95°C for 30 s, 55°C for 30 s, and 72°C for 30 s, and a final extension at 72°C for 2 min. All second PCR products were pooled in equal volumes (3 μL per sample) and purified using AMPure XP Reagent beads (Beckman-Coulter, Fullerton, CA, USA) as follows: 250 μL of pooled products and 250 μL of AMPure XP beads were mixed. After agitating for 10 min at room temperature, the sample was placed on a magnetic separator for 10 min, and the supernatant was discarded. The amplicon-containing beads were washed thrice with 500 μL of freshly prepared 80% ethanol. Finally, the purified PCR products were suspended in 50 μL of EB buffer. The quality of the amplicon library was assessed using an Agilent 2100 Bioanalyzer (Agilent Technologies, Santa Clara, CA, USA) to define the region covering all PCR library peaks (250–450 bp). The purified library was quantified using a Qubit dsDNA BR assay kit (Thermo Fisher Scientific), and then diluted to a concentration of 6 pM. Sequencing was conducted on an Illumina MiSeq system using a MiSeq Reagent Nano Kit v2 (300 cycles) with 30% PhiX control spiked in. The sequencing adaptor sites were removed, and the barcodes were demultiplexed to separate the different samples using the Local Run Manager. Following quality control by fastp, the resulting FASTQ sequences of each sample were aligned to the above reference sequence using bwa. The SNP variants of the target sites were detected with GATK version 4.4.0.0, yielding a VCF file. Then, the total number of aligned reads and those with alternative bases (ALT) in the position of interest were enumerated. The read count ratio was calculated as the ratio of the number of ALT reads to that of total reads.

### Accession number

The FASTQ sequence data used in the QTL-seq analyses are available from the DDBJ Sequence Read Archive (Accession number: DRA017689).

## Results

### Flesh color of TY, BM, and their F_1_ progenies

**Fig. 1.**
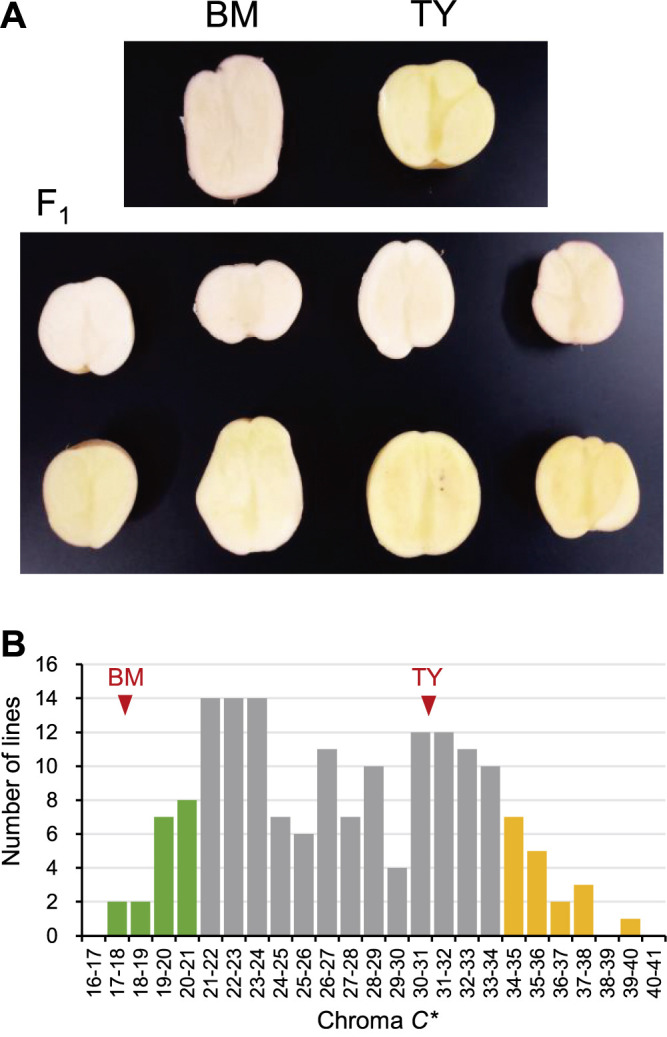
Appearance of cut surfaces of tubers and distribution of flesh color for ‘Benimaru’ (BM), ‘Touya’ (TY), and F_1_ progenies. (A) Surface appearance of tuber sections. (B) The number of F_1_ lines with specific chroma C* values determined by a color reader showing the averages for tests conducted in 2022 and 2023. Red arrowheads highlight the values of 17.78 for BM and 30.93 for TY. Green and orange represent the criteria for defining white and yellow bulks for QTL-seq analysis, respectively.

The parental cultivars BM and TY have white- and yellow-colored tubers, respectively, with BM occasionally exhibiting light red pigmentation at the vascular bundle (Fig. 1A). The tubers of the F_1_ progenies (*n* = 169, available lines for the measurement among 178 planted lines) varied in color, with five lines showing red pigmentation at the vascular bundle. To quantify the color, the International Commission on Illumination CIE 1976 color space values (L*, a*, and b*) were measured at the perimedullary zone between the pith and cortex using a color reader. Since yellow flesh coloration increases b* (yellow–blue, positive toward yellow) and a* (red–green, positive toward red), C* [chroma, calculated as the square root value of (a*)^2^ + (b*)^2^], which accounts for both values, was employed as an index of yellow coloration.

The flesh color of the F_1_ progenies, evaluated over two years (2022 and 2023), displayed a bimodal distribution. The lines with C* values of 26–27 were over-represented, while those with values of 29–30 were underrepresented (Fig. 1B). The first (ranging from 17–26) and second (27–40) peaks correspond to the white and yellow groups, respectively, with similar numbers of lines in each group (74 and 84, respectively). BM and TY belonged to the white and yellow groups, respectively. This distribution pattern suggests that a single dominant locus with the simplex genotype, either TY-derived yellowing allele or BM-derived whitening allele, governs flesh color.

### Polyploid QTL-seq analysis of flesh color and development of DNA markers linked to yellow flesh

A QTL analysis was conducted to elucidate the genetic determinants underlying flesh pigmentation. The white and yellow flesh bulks were generated using 19 lines with C* values below 21 and 18 lines with values above 34, corresponding to 11.2% and 10.7% of the population, respectively. Polyploid QTL-seq analysis utilizes variants, such as SNPs and short In/Dels (under 70 bp), uniquely residing on one (simplex) or two (duplex) of the four homoeologous chromosomes in potato, to locate the genomic regions linked to the phenotype by setting the parameter of SNP-index range for their donor parent. In this case, it was set to 0.10–0.36 or 0.37–0.63 for simplex and duplex variants, respectively, since 95% of variants with their sequencing depths of >40 are included within these ranges according to a simulation assessment (Supplemental Fig. 1). Progenies resulting from a cross between a parent possessing a simplex variant and another parent devoid of the polymorphism, referred to as a nulliplex, segregate the nulliplex and simplex genotypes at a 1:1 ratio. For duplexed variants, the ratio is 1:4:1 for nulliplex, simplex, and duplex in the progenies. In the analyses with TY-derived variants, 789,078 simplex and 212,097 duplex variants were examined, while 893,676 simplex and 284,413 duplex BM-derived variants were analyzed (Supplemental Table 1).

In the analysis involving simplex variants, the SNP-indices for simplex and nulliplex variants are 0.25 and 0, respectively. The expected SNP-index for both progeny bulks in regions unrelated to the target genes should be approximately 0.125 due to unbiased segregation of the simplex and nulliplex at a 1:1 ratio, although this value might differ for individual variants. Therefore, the ΔSNP-index, calculated by subtracting the SNP-index of the white flesh bulk from that of the yellow flesh bulk, should be around 0. However, the presence of a QTL within a region skews the SNP-index for one bulk toward 0.25 due to the predominance of simplex progenies, while the SNP-index of the other bulk approaches 0 due to an increased number of nulliplex progenies for the homoeologous chromosome containing the causative gene. This either elevates or reduces the ΔSNP-index from 0 to 0.25 or –0.25. Even in the case that the distribution of one homoeologous chromosome is skewed, the transfer of the other chromosomes, devoid of the causative gene, to the progenies occurs without any selection. The SNP-index for the variants on those chromosomes is typically around 0.125 for both bulks, resulting in numerous dots of the ΔSNP-index hovering around 0. Due to the stochastic reading of homoeologous chromosomes, SNP-indices might differ from the expected value, resulting in certain SNPs exhibiting index values surpassing statistical confidence thresholds in regions with a vast number of SNPs. In these regions, numerous ΔSNP-index dots are dispersed symmetrically above the upper threshold and below the lower threshold. However, in the presence of a QTL, the SNP-index of one bulk is elevated while it is close to 0 for the other, causing the ΔSNP-index values to exceed the threshold solely on one side. Considering such an asymmetrical distribution of ΔSNP-index dots, QTL candidate regions were identified when the P99 + or – ΔSNP-index QTL variant count, which is the number of QTL-deduced variants with positive or negative ΔSNP-index by the P99 criteria in the 100-kb sliding window (listed in the SlidingWindow.txt file provided as an output file of the polyploid QTL-seq pipeline), was beyond 100 in one direction of QTL effect but not in the other direction. In this study, the threshold value was set to 100 by considering the number of P99 variants throughout all chromosomes.

**Fig. 2.**
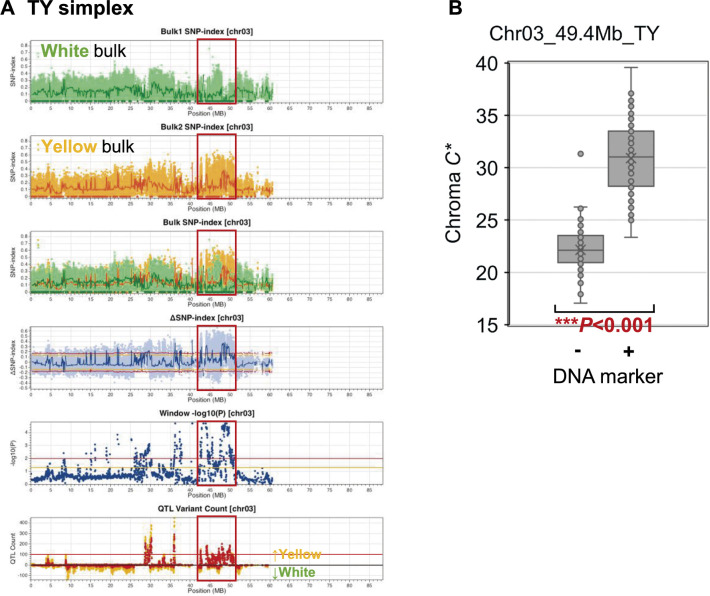
The genomic region associated with yellow flesh color in F_1_ progenies. (A) Polyploid QTL-seq analysis employing TY-derived simplex variants. SNP-index plots of the white and yellow bulk, their superimposed plot, ΔSNP-index plot, window –log_10_*P* plot, and QTL variant count plot are depicted. Dark green and orange dots in the SNP-index plots denote variants with an SNP-index of 0. Green, red, and blue lines represent the sliding window average of a 100 kb interval with a 20 kb increment for SNP-index and ΔSNP-index. The window –log_10_*P* plot displays the average of the –log_10_*P* values of all variants within the sliding window. Orange and red lines on the ΔSNP-index plot and the window –log_10_*P* plot represent 95% and 99% statistical confidence thresholds under the null hypothesis of no QTLs, respectively. The QTL variant count plot illustrates the number of QTL-deduced variants in the sliding window. Orange and red dots on the QTL variant count plot indicate the number of variants deduced as QTL with 95% and 99% statistical confidence, respectively, while red lines signify the threshold of 100 for determining the QTL candidates. Variants are categorized based on the direction of QTL effects, with those exhibiting positive and negative ΔSNP-index values plotted upward and downward, respectively, on the QTL variant count plot. A candidate region identified as QTL is delineated by red frames. The graphs are depicted for Chr03 in the TY-derived simplex analysis. Additional chromosomes and those for the analyses with duplex variants are presented in Supplemental Fig. 2. (B) Genotyping using a developed DNA marker. Assessment of a flesh color-associated SNP marker derived from the TY simplex variant (Chr03_49.4Mb_TY) was conducted. The association between the marker genotype (+; presence or –; absence) and flesh color chroma C* in the F_1_ population (*n* = 169) is depicted. The box plots illustrate the minimum, first quartile, median, third quartile, and maximum values, with the “x” marks representing the mean values. Significant differences are denoted by asterisks (Student’s *t*-test, ***P < 0.001). Amplification using control *GBSS* primers was validated.

Applying these criteria, the regions spanning 28.6–30.3 Mb, 35.8–36.1 Mb, and 42.5–50.0 Mb on Chr03 were analyzed using TY-derived simplexes (Supplemental Table 3). Although the reason why the sharp peaks appear within narrow regions such as 2 Mb is unknown, repetitive recombination does not frequently occur within such narrow intervals. Therefore, the former two regions are unlikely to be QTLs. The region of Chr03, 42.5–50.0 Mb, was considered the most likely QTL candidate. Within this region, the yellow flesh bulk exhibited SNPs with elevated SNP-indices compared to that of the white flesh bulk (Fig. 2A, see upper three graphs). Notably, an asymmetrical distribution of ΔSNP-index dots was observed predominantly exceeding the upper threshold in the positive direction (Fig. 2A, the fourth graph from top), implying that this QTL likely increases yellow pigmentation. Furthermore, the white flesh bulk sample displayed a huge SNP cluster with an SNP-index of 0 (Fig. 2A, the uppermost graph, Supplemental Table 3). In this region, the number of P99 variants exceeds 100 (Fig. 2A, two graphs from bottom). However, significant candidates were not identified on the other chromosomes and on those with BM-derived simplexes or duplexes derived from both parents (Supplemental Fig. 2, Supplemental Tables 3–5).

After identifying the candidate region for yellow flesh color, a linked PCR marker was developed at an SNP site in the middle of the region (base 49,441,219) on Chr03, where the SNP-indices were 0 and 0.259 for white and yellow flesh bulk, respectively (Supplemental Table 4). PCR and agarose gel electrophoresis analyses showed that this marker, Chr03:49441219/C, targeted the TY-specific T to C SNP and yielded a distinctive fragment in TY but not in BM. The 169 F_1_ progenies were genotyped (Supplemental Fig. 3). The individuals carrying the TY-derived allele (Chr03_49.4Mb_TY) displayed significantly higher C* values than those without, dividing them into white and yellow flesh groups (Fig. 2B), although one progeny presented a high C* value even in the absence of the allele exceptionally. This confirmed that the TY-derived simplex allele in this locus solely governs yellow pigmentation of tuber flesh.

### Starch phosphorus content of TY, BM, and their F_1_ progenies

**Fig. 3.**
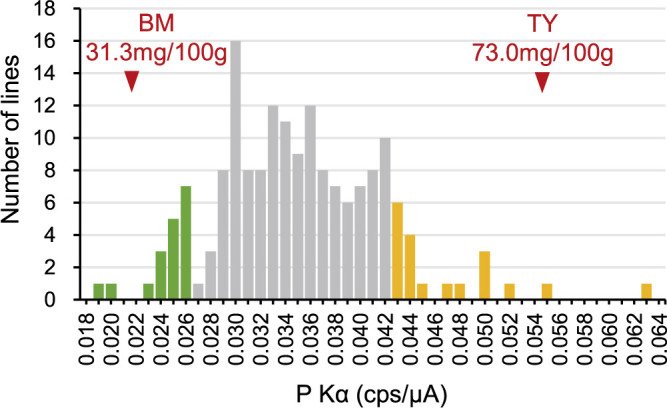
Tuber starch phosphorus content of BM, TY, and F_1_ progenies. The number of F_1_ lines with specific P Kα count determined by energy-dispersive X-ray fluorescence spectroscopy and the averages for tests conducted in 2022 and 2023 are presented. Red arrowheads indicate the values of 0.0215 cps/μA for BM and 0.0545 cps/μA for TY, along with the phosphorus contents determined by inductively coupled plasma-optical emission spectrometry, 31.3 and 73.0 mg/100 g, respectively. Criteria for low and high bulks for QTL-seq analysis are denoted in green and orange, respectively.

The phosphorus content of tuber starch, analyzed by P Kα of energy-dispersive X-ray fluorescence spectroscopy, has been shown to be proportional to phosphorus content in potato starch (Noda *et al.* 2006a). Absolute phosphorus contents of BM and TY were 31.3 and 73.0 mg/100 g starch, respectively, as determined by inductively coupled plasma-optical emission spectrometry. They exhibited 0.0215 and 0.0545 cps/μA, respectively. The F_1_ progenies (*n* = 171, available lines for the measurement among 178 planted lines) displayed a wide range of values from 0.0190 to 0.0630 cps/μA, with an average of 0.0353 cps/μA (Fig. 3), suggesting that starch phosphorus content is governed by multiple loci or multiplexed alleles.

### Polyploid QTL-seq analysis of starch phosphorus content and development of DNA markers linked to low phosphorus starch

The QTLs related to starch phosphorous content were analyzed using 18 lines with P Kα values <0.0265 cps/μA and 16 lines with >0.0435 cps/μA, corresponding to 10.5% and 9.4% of the population, to generate low and high phosphorus bulks, respectively. For the analyses with TY-derived variants, 780,645 simplex and 206,603 duplex variants were exploited, while 883,470 simplex and 277,314 duplex variants were subjected to the analyses with BM-derived variants (Supplemental Table 1).

**Fig. 4.**
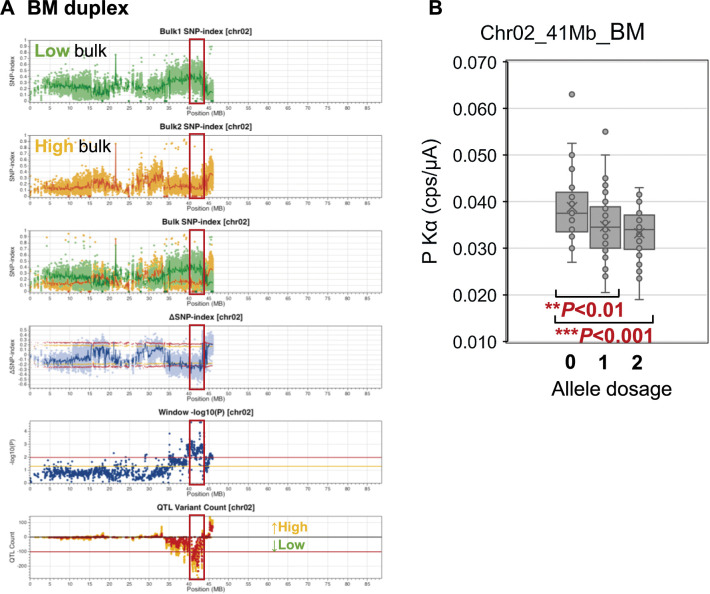
The genomic region associated with starch phosphorus content in F_1_ progenies. (A) Polyploid QTL-seq analysis using BM-derived duplex variants. Plots consisting of the SNP-index of low bulk and high bulk, their superimposition, ΔSNP-index, window –log_10_*P*, and QTL variant counts are depicted, as shown in Fig. 2. Red frames indicate candidate regions deduced as QTLs. The graphs are depicted for Chr02 in the BM-derived duplex analysis. Additional chromosomes and those for the analyses with simplex variants are presented in Supplemental Fig. 4. (B) Genotyping using developed DNA markers. Evaluation of phosphorus content-linked SNP markers from the BM duplex variants (Chr02_41Mb_BM). The relationship between the allele dosage (0; nulliplex, 1; simplex, and 2; duplex) and the P Kα value is shown for the F_1_ population (*n* = 171). The box plots illustrate the minimum, first quartile, median, third quartile, and maximum values, with the “x” marks representing the mean values. Asterisks denote significant differences (**P < 0.01, ***P < 0.001), as examined by ANOVA.

Applying the same criteria for QTL identification as above, the analysis with BM-derived duplex variants identified a sole candidate region from 39.7 to 43.4 Mb on Chr02 (Supplemental Table 6). Within this region, the low phosphorus bulk exhibited SNPs with higher SNP-indices compared to the high phosphorus bulk (Fig. 4A, upper three graphs). An asymmetrical distribution of ΔSNP-index dots was predominantly observed below the lower threshold in the negative direction (Fig. 4A, the fourth graph from top) along with the number of P99 variants surpassing 100 (Fig. 4A, two graphs from bottom), indicating the presence of a QTL associated with decreased phosphorus content. However, the analysis could not detect significant candidates on the other chromosomes. Furthermore, no other candidates were identified in the analysis with BM-derived simplexes, TY-derived simplexes and TY-derived duplexes, except for those occurring at a single section of sliding window (Supplemental Fig. 4, Supplemental Tables 6–8).

Since the above candidate regions were identified in the analysis using duplex variants retained in BM, the genotypes of the 171 F_1_ individuals were investigated using plexity-distinguishable genotyping methods, such as quantitative PCR, fragment analysis, and amplicon sequencing.

For quantitative PCR, a Chr02:41426723/A marker was created targeting the BM-specific G to A SNP on Chr02 at base 41,426,723 (Supplemental Table 2), where SNP indices were 0.506 and 0.070 for low and high phosphorus bulk, respectively (Supplemental Table 8). This resulted in efficient amplification with BM harboring the intended allele in the duplex but not with TY, which is devoid of it. Contrastingly, partial amplification was achieved with a mixture of an equal amount of BM and TY DNA, which mimics the allele dosage of simplex (Supplemental Fig. 5A). When the allele dosage of the mixture assuming simplex was set to 1.0, those of TY and BM were 0.02 and 1.84, respectively (Supplemental Table 9). Distribution in allele dosage of the F_1_ progenies, as calculated with the Ct value of each sample, presented three discernable subgroups of the allele dosage of 0–0.1, 0.4–1.5, 1.5–2.2, corresponding to nulliplex, simplex, and duplex genotypes, respectively.

Fragment analysis was also used to perform genotyping using a set of competitive markers, Chr02:41523385/A_ALT and Chr02:41523385/T_REF, targeting the BM-specific T to A SNP on Chr02 at base 41,523,385 (Supplemental Table 2). The SNP-indices were 0.471 and 0.063 for low and high phosphorus bulk, respectively (Supplemental Table 8). Those markers were applied in a single PCR. Chr02:41523385/A_ALT and Chr02:41523385/T_REF yielded 135 bp and 138 bp amplicons for reference (REF)-type T and alternative (ALT)-type A alleles, respectively, in an allele dosage-dependent manner (Supplemental Fig. 5B). Since two DNA primers for distinguishment of the REF-type and ALT-type alleles differ in a single base at their 3ʹ terminal, a small amount of fragment would be amplified from the REF-type allele with the ALT-type primer. Therefore, a small ALT-peak was observed for the nulliplex sample. Taking the ratio of the height of their peaks in the electropherogram, the ratios of TY, the mixture of both parents assuming simplex, and BM were 0.123, 0.347, and 0.718, respectively (Supplemental Table 9). The F_1_ progenies were distributed into three distinct subgroups corresponding to nulliplex (0.05–0.15), simplex (0.25–0.55), and duplex (0.55–1.15). The border between simplex and duplex was not obvious but divided in the middle by 0.55.

Finally, the genotypes were confirmed by amplicon sequencing with three primer sets, Chr02:41322042, Chr02:41426723, and Chr02:41523385, targeting the BM-specific G to A SNP on Chr02 at base 41,322,042, G to A SNP on Chr02 at base 41,426,723, and T to A SNP on Chr02 at base 41,523,385 (Supplemental Table 2) with SNP-indices of 0.476 and 0.071, 0.506 and 0.070, and 0.471 and 0.063 for low and high phosphorus bulk, respectively (Supplemental Table 8). For Chr02:41322042, the BM-specific SNPs were detected with the read count ratio of 0.002, 0.232, and 0.487 in TY, the mixture of both parents assuming simplex, and BM, respectively. Similarly, the ratios in Chr02:41426723 and Chr02:41523385 were 0.004, 0.240, and 0.545 and 0.000, 0.214, and 0.475, respectively (Supplemental Table 9). These ratios aligned with the SNP-indices for both parents in the QTL-seq analysis. Distribution of the F_1_ progenies showed discrete subgroups for nulliplex (0.00–0.025), simplex (0.150–0.375), and duplex (0.375–0.700) in all the amplicons (Supplemental Fig. 5C).

The plexities suggested by the above three methods were compared (Supplemental Table 9). The genotypes of 162/178 lines (91%) were consistent throughout all analyses. In the 16 lines (9%) that showed a discrepancy in at least one marker, most frequently suggested genotype was considered as the genotype of the line. The ratio of individuals with nulliplex, simplex, and duplex genotypes was 39:101:38, which was slightly different from 29.7:118.7:29.7, the expected ratio for the segregation of F_1_ progenies generated by the cross between duplexed and nulliplexed parents (*P* = 0.020 as examined by *χ*^2^ test). Among them, the starch phosphorus content in the tubers of 171 progenies was analyzed. The individuals carrying the BM-derived allele (Chr02_41Mb_BM) displayed significantly lower P Kα values than those lacking this allele in an allele dosage-dependent manner (Fig. 4B), confirming that the BM-derived allele in this locus mainly determines the decrease in starch phosphorus content.

## Discussion

Potato, an autotetraploid plant, consists of four sets of homoeologous chromosomes, and two are randomly inherited from each parent to the progenies. This complex mode of chromosome inheritance, along with the high similarity in nucleotide sequences among the four sets, poses challenges for the genetic dissection of agronomic traits using conventional analytical methods designed for diploid crops. Previously, we developed a polyploid QTL-seq program, enabling the rapid identification of genomic regions associated with agronomic traits in polyploid crops, such as sweetpotato flesh texture (Yamakawa *et al.* 2024). Although this method performs QTL mapping using extracted simplex sequence variants exclusively located on single homoeologous chromosomes, it can only be used to explore QTLs with simplex genotypes. The polyploid QTL-seq program itself is capable of surveying QTLs with non-simplex genotypes, such as duplex, by adjusting parameters to select variants used for analyses. Here, we verified the detection of such multiplexed QTLs in the F_1_ progenies derived from a cross between BM (white flesh and low starch phosphorus content) and TY (yellow flesh and high starch phosphorus content) potatoes. By employing settings for selecting simplex and duplex, wherein variants with SNP-indices of 0.10–0.36 and 0.37–0.63 were used for QTL mapping, respectively, we identified a single QTL responsible for yellow flesh color on Chr03 in simplex (Fig. 2) and another QTL for low starch phosphorus content on Chr02 in duplex (Fig. 4). The distribution of both flesh color and starch phosphorylation in F_1_ population was continuous. Therefore, involvement of more than one QTLs was expected. However, only one QTL was identified for both traits. These traits might be affected by environmental factors such as temperature. Consistently, increase of starch phosphorus content by late harvest was reported (Noda *et al.* 2004). Furthermore, we developed allele-specific DNA markers linked to these effective QTLs (Supplemental Tables 2, 9). Notably, plexity-distinguishable markers were established for the duplex QTLs of starch phosphorus content using various methods, including quantitative PCR, fragment analysis, and amplicon sequencing (Supplemental Fig. 5).

The yellow pigments in tetraploid potato mainly comprise lutein and violaxanthin (Kobayashi *et al.* 2008, Morris *et al.* 2004, Nesterenko and Sink 2003). The alleles (*Bch*, *Chy2*) for β-carotene hydroxylase, the enzyme responsible for their biosynthesis, have a dominant effect on changing white to yellow flesh color (Brown *et al.* 2006, Wolters *et al.* 2010). Consistently, a major QTL for yellow flesh color was previously identified at the β-carotene hydroxylase locus on Chr03 in diploid segregating populations (Campbell *et al.* 2014, Endelman and Jansky 2016, Hara-Skrzypiec *et al.* 2018, Meijer *et al.* 2018) and tetraploid GWAS populations (D’hoop *et al.* 2014, Pandey *et al.* 2022, Uitdewilligen *et al.* 2013). Moreover, the expression level of the β-carotene hydroxylase gene has been associated with yellow flesh color in the progenies of a diploid segregating population (Kloosterman *et al.* 2010). In the present study, a single major QTL was detected at Chr03 (Fig. 2). Therefore, we speculated that the yellow flesh parent, TY, harbors an active β-carotene hydroxylase gene, while the white flesh parent, BM, does not. In the reference genome sequence of potato *S. tuberosum* Group Phureja DM 1-3 516 R44 v6.1 (Pham *et al.* 2020), the β-carotene hydroxylase gene (Soltu.DM.03G018410) resides at 42.89 Mb. Within its coding region, three QTL-deduced SNPs, all C to T conversions, were detected at the bases 42,893,787, 42,895,229, and 42,895,430 (Supplemental Table 4). The first one leads to the conversion of the 312nd Glu to Gly of the 314 amino-acid peptide, while the latter two are a mutation in the 2nd intron and a silent mutation on the 128th Ser, respectively (Supplemental Fig. 6A, 6C). Neighboring SNPs closest to the coding region were detected 7.3 kb upstream and 1.4 kb downstream of the coding region. Furthermore, manual inspection of the mapped reads revealed 10 more TY-specific SNPs in the intron regions [G to A at base 42,894,053, A to C at base 42,894,069, A to G at base 42,894,205 (one base upstream of splicing junction “AG”), C to T at base 42,894,235, G to A at base 42,894,252, C to A at base 42,894,286, T to C at base 42,894,568, A to T at base 42,895,073, A to G at base 42,895,097, and A to C at base 42,895,373] are inherited exclusively to the yellow flesh bulk (Supplemental Fig. 6A). However, such SNPs were not found within 250 bp upstream of the transcript region. The effects of the amino acid conversion at the C-terminal region on the enzyme’s activity and of the mutations in the introns on the transcription efficiency remain undetermined. However, the flesh color is evidently governed by a single locus on Chr03 in the progenies generated by the cross between TY and BM.

A high content of covalently bound phosphate is a unique characteristic of potato starch. In a previous QTL study with a mapping population of diploid potatoes, genomic regions associated with starch phosphorus content were detected at Chr02, Chr05, and Chr09 (Werij *et al.* 2012). Among them, the genes for starch synthase II (*SSII*) and α-glucan water dikinase (*GWD*) co-localized on the QTL regions of Chr02 and Chr05, respectively. Furthermore, a GWAS study using 193 tetraploid potato lines identified SNPs and SSRs associated with starch phosphorus contents on *GWD*, starch branching enzymes I and II (*SBEI* and *SBEII*), and starch synthase III (*SSIII*) (Carpenter *et al.* 2015). The deletion of GWD, which phosphorylates α-glucan chains in starch (Van Harsselaar *et al.* 2017), results in the reduction of starch phosphate (Ohnuma *et al.* 2023, Wickramasinghe *et al.* 2009). Furthermore, allelic variation for *GWD* is associated with starch phosphate content in potato (Uitdewilligen *et al.* 2022). SBE is an enzyme that attaches short α-glucan chains onto the existing amylopectin chains in starch, creating a highly branched structure (Nakamura 2002, Van Harsselaar *et al.* 2017). Its defect yielded starch with increased phosphorus contents (Jobling *et al.* 1999, Safford *et al.* 1998, Wickramasinghe *et al.* 2009). The starch phosphorus content in transgenic potatoes devoid of SSII and SSIII, enzymes that elongate α-glucan chains on amylopectin in starch (Nakamura 2002, Van Harsselaar *et al.* 2017), were reduced (Jobling *et al.* 2002), indicating its involvement in starch phosphorylation. This effect is possibly caused by the decrease of the amylopectin chain length, reducing the surface that phosphorus can attach to. In the present study, a single duplex QTL was detected at 39.7–43.4 Mb on Chr02 (Fig. 4) in the vicinity of *SSII* (Soltu.DM.02G031690), located at 43.71 Mb. *SSII* is not included in the candidate region due to a small number of 91 polymorphisms within the 200-kb region covering 100 kb upstream and downstream of the gene (Supplemental Table 8). However, 53 P99– variants were detected in the region, whereas no P99+ variants existed. Therefore, we speculated that *SSII* is the candidate gene responsible for the changes in starch phosphorus content. The BM-specific *SSII* allele included a single base insertion at base 43,711,865 (Supplemental Table 8), leading to a frameshift mutation immediately before the stop codon. Consequently, the enzyme encoded by this allele contains a 49 amino acid extension on its C-terminal (Supplemental Fig. 6B). According to SWISS-MODEL, this extension is estimated to protrude in the opposite direction of a catalytic groove (Supplemental Fig. 6D). However, its possible inhibitory effect on the activity by intramolecular and intermolecular conformational interference should be considered as the 49 amino-acid peptide was not incorporated in the modeling because of a lack of similar peptides in the SWISS-MODEL database. In cereal endosperm, starch biosynthetic enzymes are reported to form multi-protein complexes (Crofts *et al.* 2017, Ida *et al.* 2022). It might be possible that the BM-derived SSII allele with the C-terminal extension have dominant-negative effect by interfering the formation of active complex of starch synthase. If the BM-derived SSII has reduced activity, the difference in phosphorus content can be explained by the dosage of the extension-free active allele. BM has two extension-free alleles, whereas TY has four. Whether the BM-specific SSII with the extension has reduced activity or not, its effect on the starch phosphorus content remains to be determined.

Three types of DNA markers were developed to distinguish the dosage of the BM-specific allele at the QTL region associated with starch phosphorus content. Although quantitative PCR, a simple, straightforward method for determining allele dosage, requires real-time PCR equipment, it is the primary choice. In the present study, an analysis with an equal quantity of DNAs as templates achieved stable amplification to distinguish the difference of plexities as the DNA was sufficiently purified by extraction with a silica membrane-bound purification kit. During selection for practical breeding purposes, DNA would be extracted using simple procedures, such as ethanol precipitation of saline-buffered extracts. As crude extracts might hamper stable amplification, internal control primer sets like those of *GBSSI* would be necessary to correct differences in DNA amounts among samples. Otherwise, KASP markers would be an alternative in which PCR products corresponding to respective alleles are amplified competitively with different fluorescent-labeled primer sets to distinguish two types of bases at the targeted SNP site. Using a KASP marker, Asano and Endelman (2024) determined the plexities of a *potato virus Y* resistance gene. Fragment analysis is a second choice, although it requires a capillary DNA sequencer. Once competitive pair of allele-specific primers are established to recognize two different bases at the SNP site, a single tube amplification and subsequent electrophoresis by the sequencer can enable rapid determination of sample plexities. Amplicon sequencing would be the most accurate method as common primer sets are used to amplify both alleles. The targeted SNP sites in the amplified regions hardly affect the primer’s annealing efficiency. However, it requires a short read DNA sequencer and complicated sample handling to manipulate different sets of index primers corresponding to each sample. All these methods yielded reliable genotyping results and revealed that over 91% of the lines had consistent genotypes (Supplemental Table 9). Overall, quantitative PCR including KASP and fragment analysis can be applied to the on-site selection of breeding materials. However, amplicon sequencing would be the additional method in cases where an accurate determination is needed.

In conclusion, we verified the polyploid QTL-seq method to explore multiplex QTLs in tetraploid potato. Since it is difficult to estimate allele-dosage of the target gene prior to the construction of the analytical population, detection of such non-simplex QTLs can expand the possibility of genetic analysis in the polyploid crops. We identified simplex and duplex QTL for yellow flesh color and starch phosphorus content, respectively. Notably, the latter QTL was not detected when simplex variants were used solely, but incorporating duplex variants enabled its detection. Since the QTL-seq method is based on whole-genome sequencing, numerous SNPs are presented in the QTL regions. Therefore, various linked DNA markers can be designed even in cases where plexities must be distinguished. The pipeline can be accessed at GitHub (https://github.com/TatsumiMizubayashi/PolyploidQtlSeq). This method can be applied to various polyploid crops to advance genome breeding.

## Author Contribution Statement

HY conceived the research project and designed the experiments. ES, K. Asano, K. Akai, and KK created the potato F_1_ population. TN developed starch phosphorus quantification methods. TM developed the polyploid QTL-seq pipeline. HY and UY conducted the quantitative PCR analysis, and HY and TA performed the fragment analysis. HY, NK, and YM conducted the amplicon sequence analysis. HY conducted all other experiments, polyploid QTL-seq, and DNA marker analyses and wrote the manuscript.

## Supplementary Material

Supplemental Figures

Supplemental Table 1

Supplemental Table 2

Supplemental Table 3

Supplemental Table 4

Supplemental Table 5

Supplemental Table 6

Supplemental Table 7

Supplemental Table 8

Supplemental Table 9
