# Supplementary material for: Polyploid QTL-seq identified QTLs controlling potato flesh color and tuber starch phosphorus content in a plexity-dependent manner
Source: Breed Sci. 2024 Nov 23;74(5):403–14. doi: 10.1270/jsbbs.24028 (PMC11780331; doi:10.1270/jsbbs.24028)
Supplement: Supplementary file 1 — Supplemental Figures [file 74_403_s1.pdf]

doi: 10.1270/jsbbs.24028

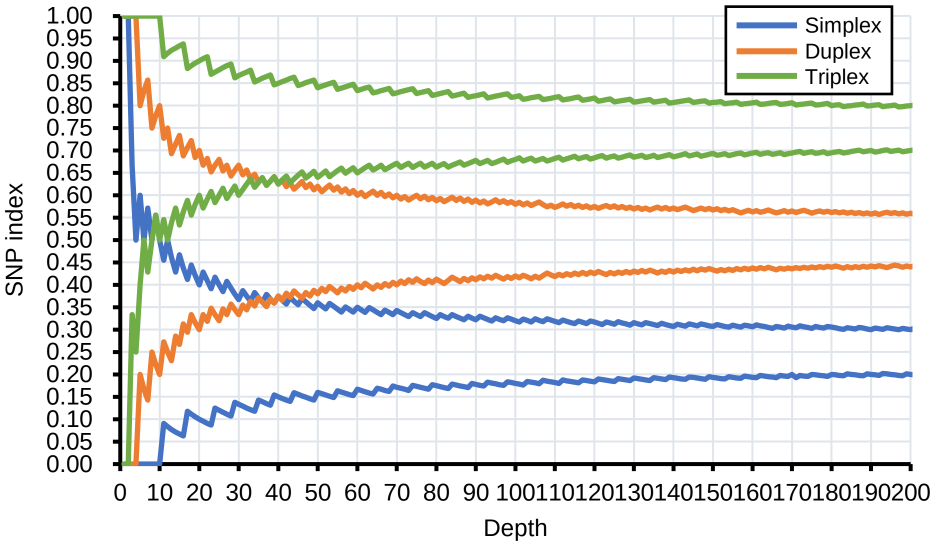

Supplemental Fig. 1. Simulation test for obtaining a 95% confidence interval assuming simplex and duplex SNPs. The 95% confidence interval of SNP index value is calculated by simulation of 10,000 replications assuming simplex (blue), duplex (orange), and triplex (green) SNPs at a given depth in case of tetraploid, potato. Lower and upper limits are indicated with lines in respective colors.

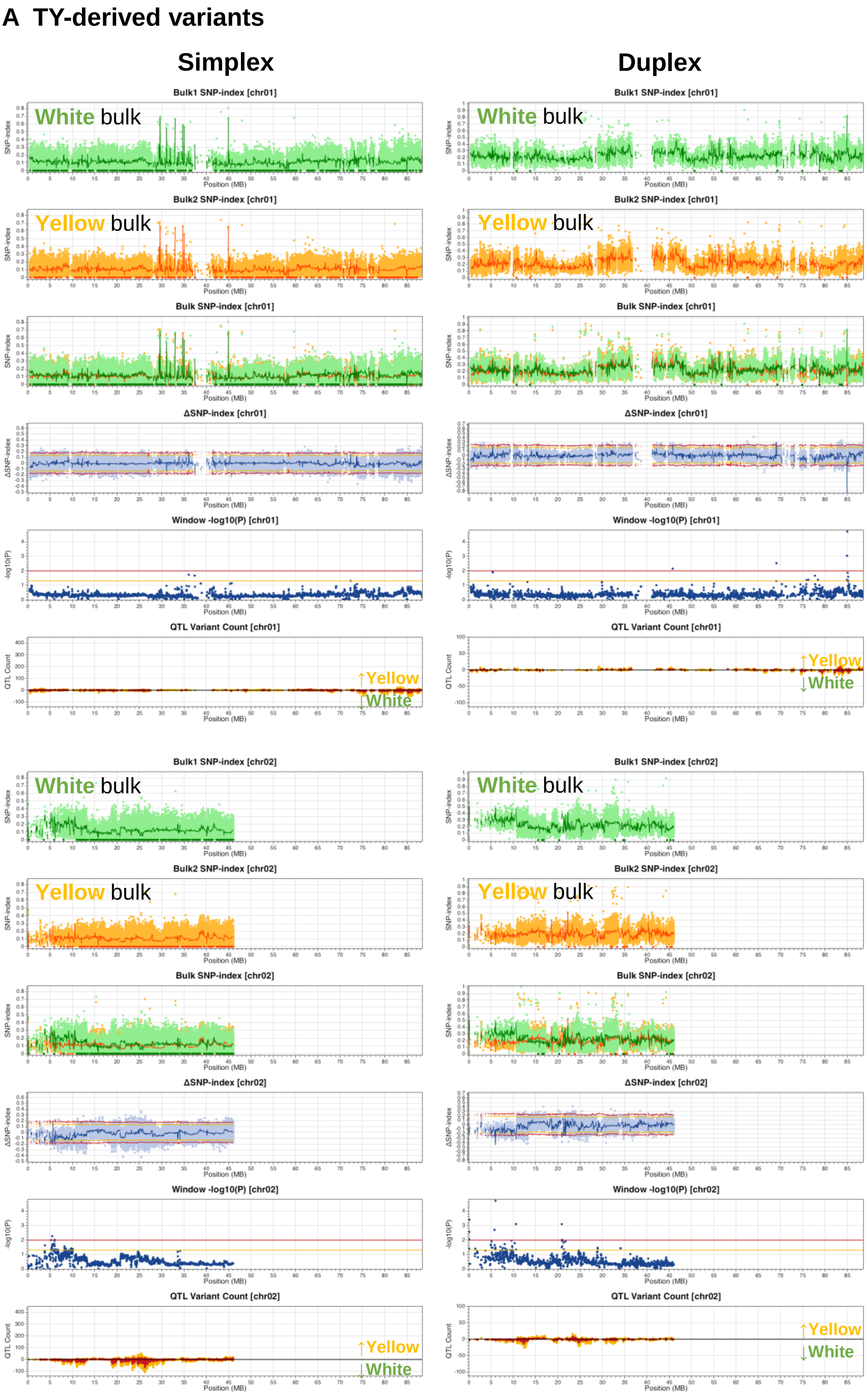

Supplemental Fig. 2. A genomic region governing yellow flesh color in F_1 progenies. (A) Polyploid QTL-seq analyses using each of TY-derived simplex and duplex variants. (B) Polyploid QTL-seq analyses using each of BM-derived simplex and duplex variants. The plots show the SNP-index of white flesh bulk and yellow flesh bulk, their superimposition, ΔSNP-index, window $-\log_{10}P$, and QTL variant count, as shown in Fig. 2. Red frames indicate a candidate region for QTL.

A TY-derived variants

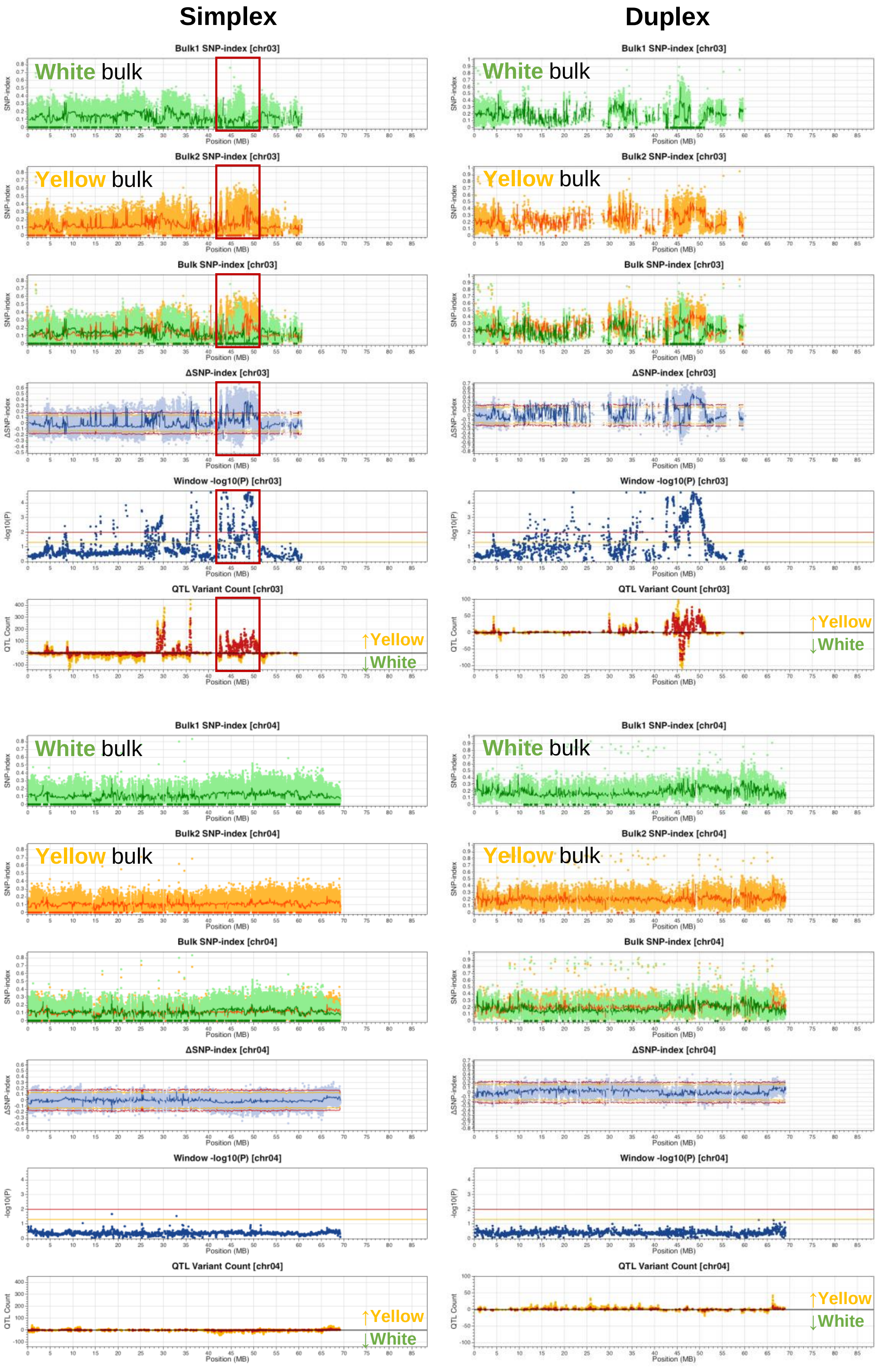

Supplemental Fig. 2. (continued)

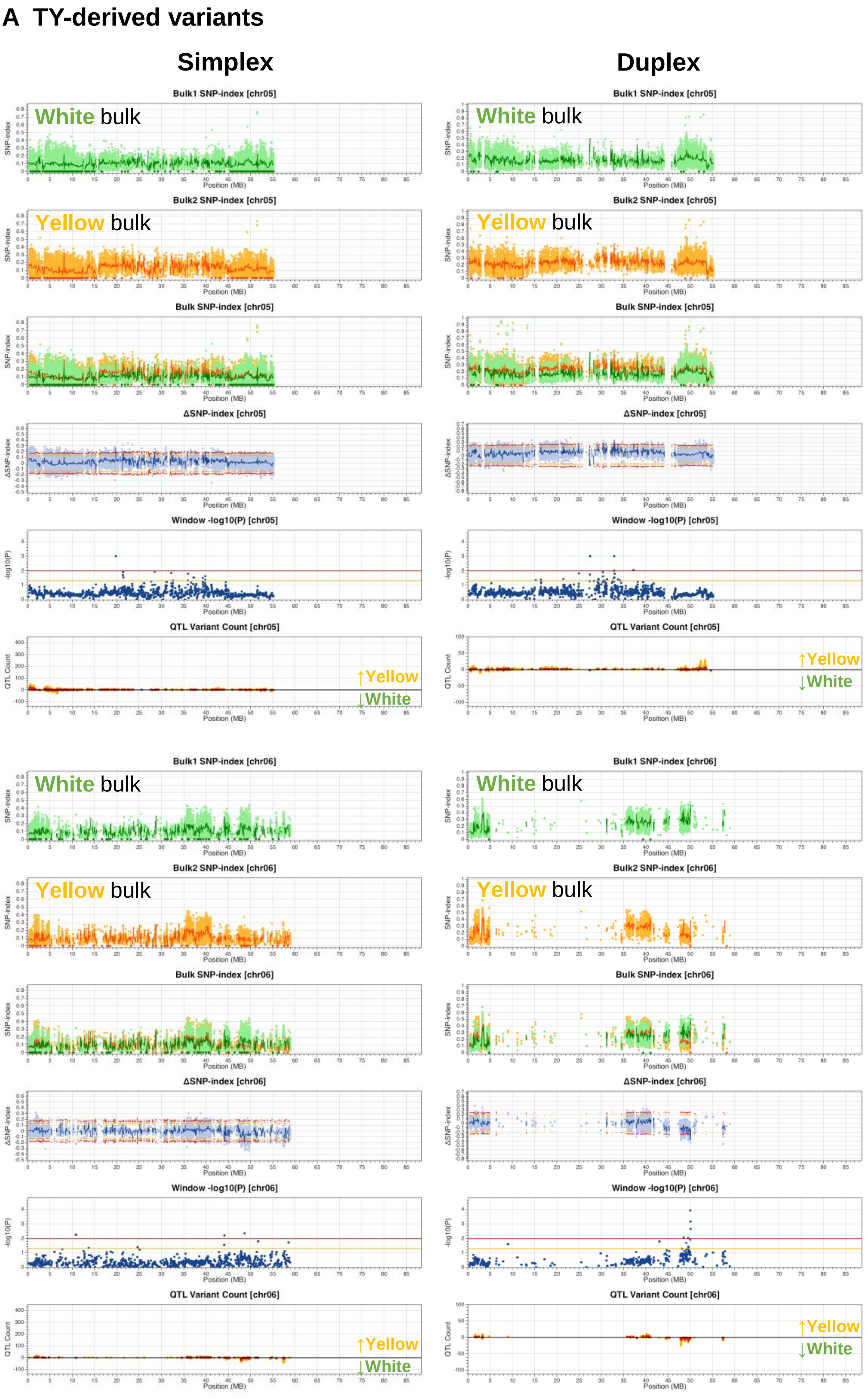

Supplemental Fig. 2. (continued)

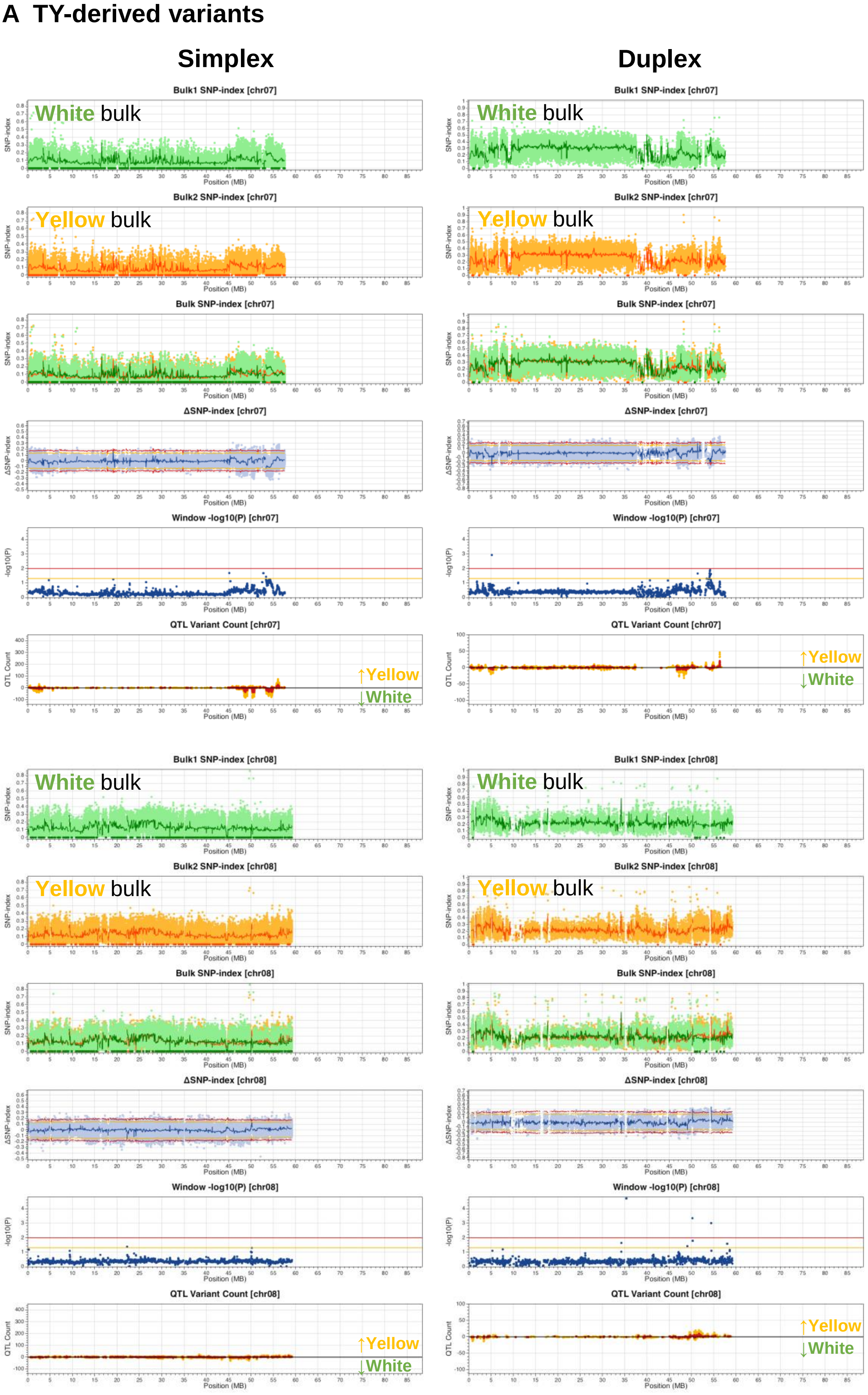

Supplemental Fig. 2. (continued)

A TY-derived variants

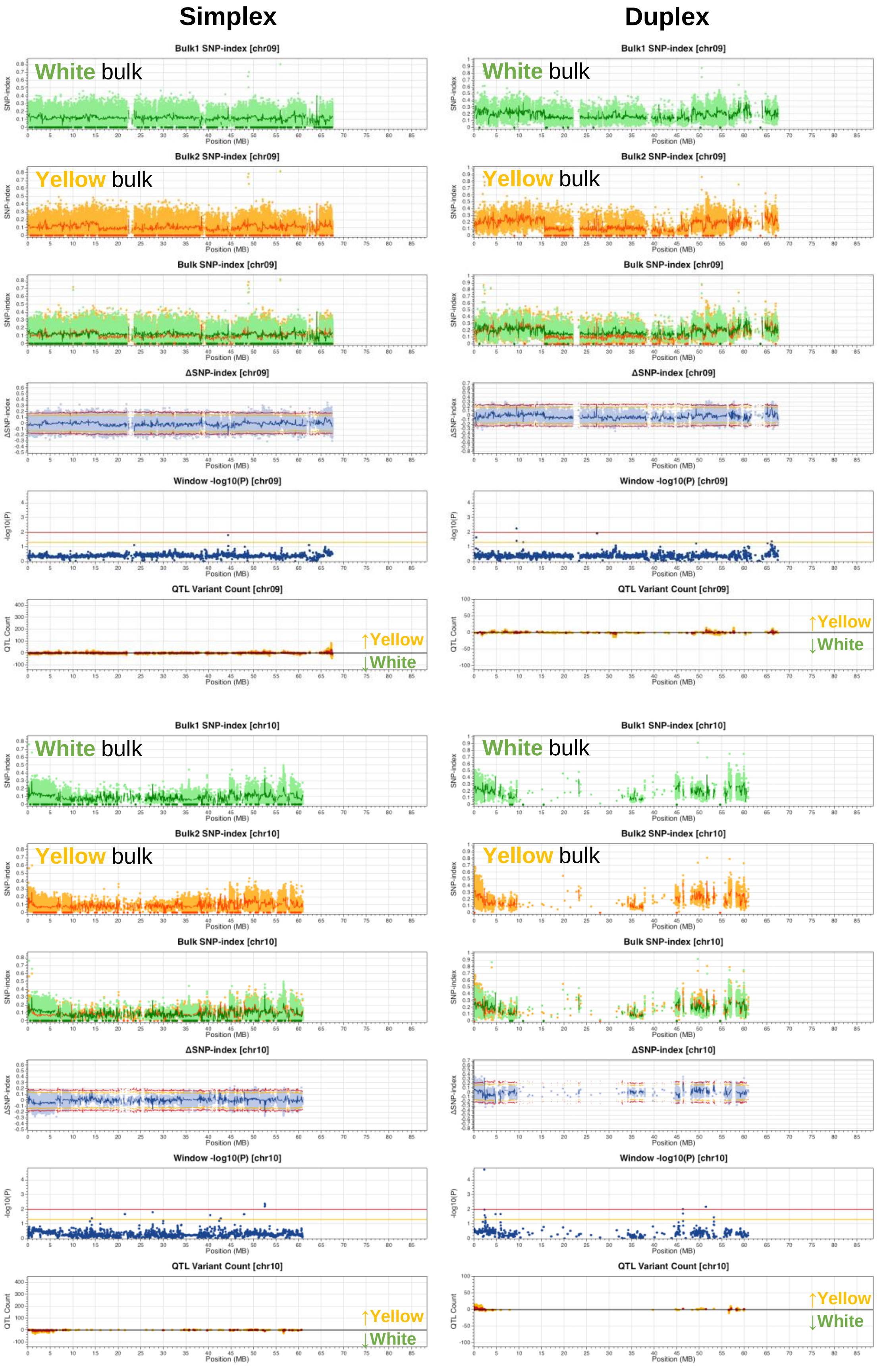

Supplemental Fig. 2. (continued)

A TY-derived variants

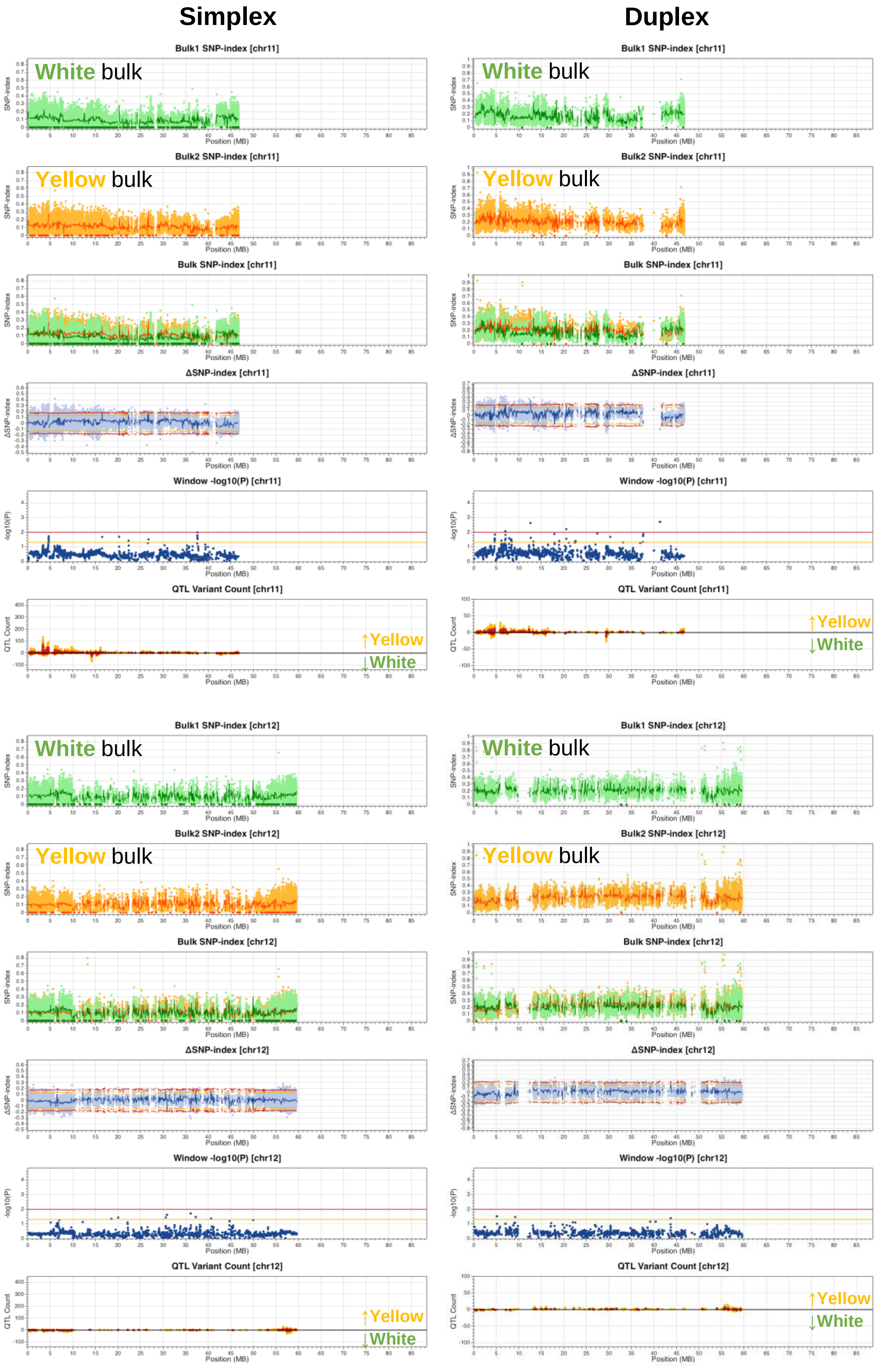

Supplemental Fig. 2. (continued)

B BM-derived variants

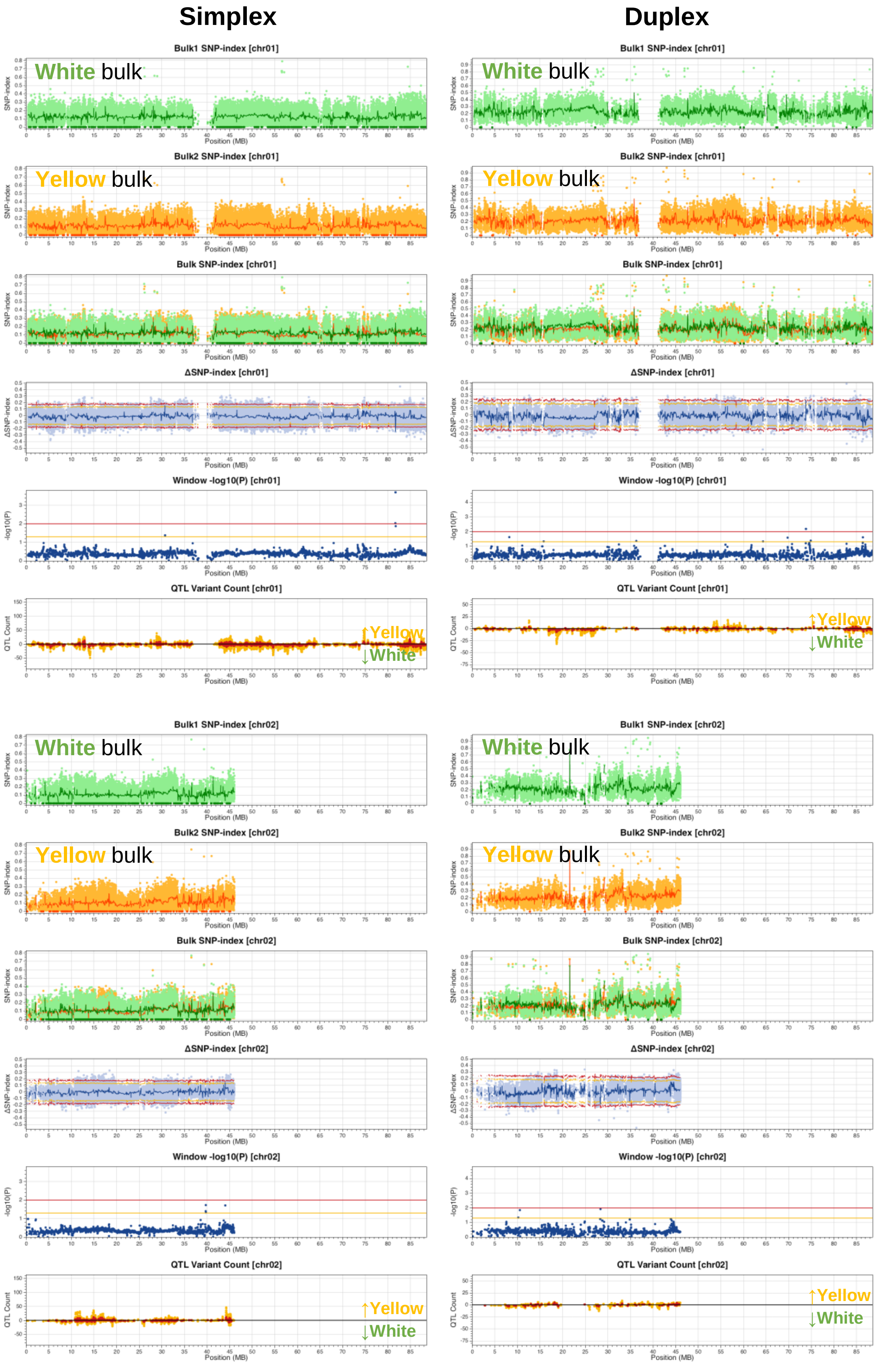

Supplemental Fig. 2. (continued)

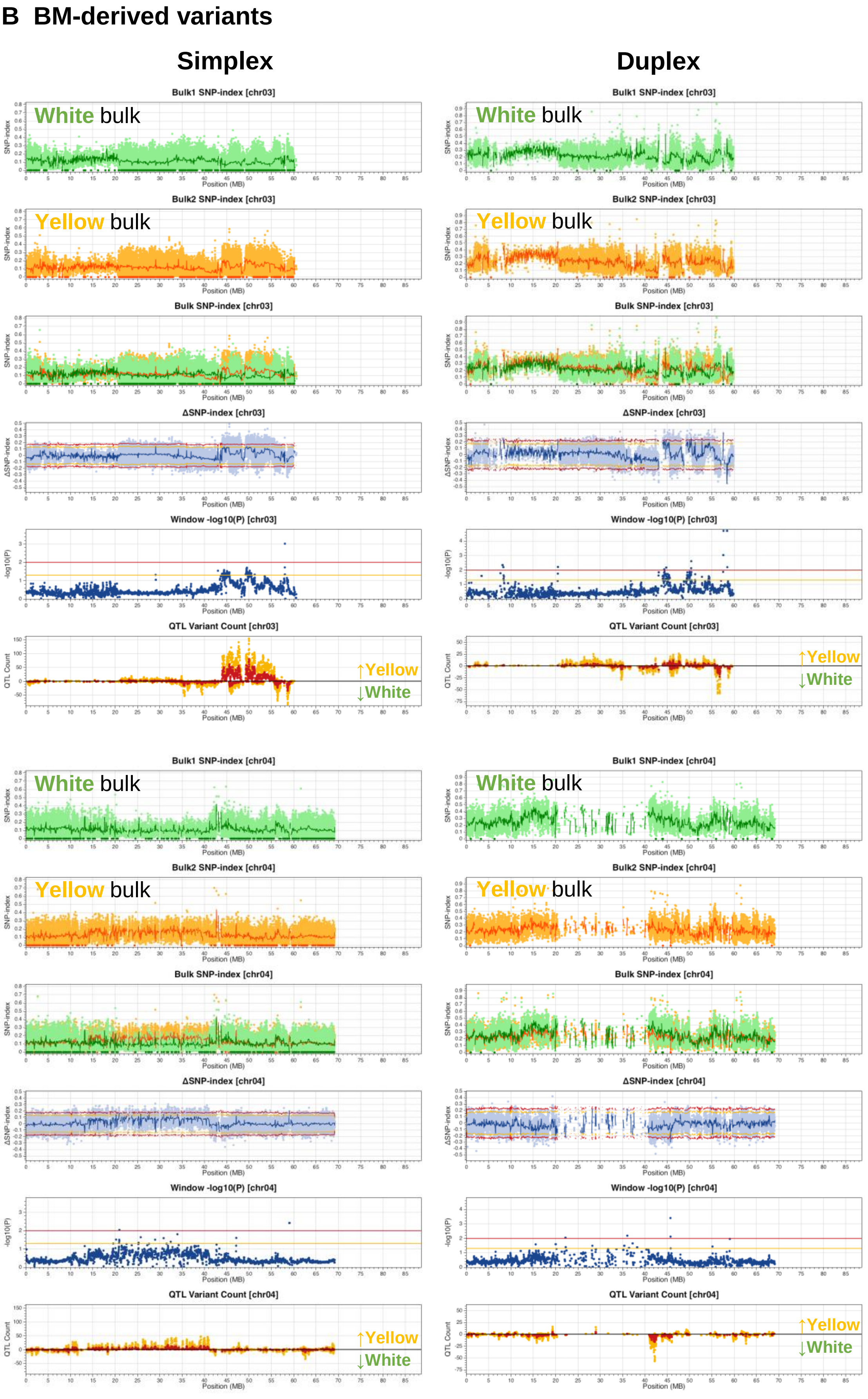

Supplemental Fig. 2. (continued)

B BM-derived variants

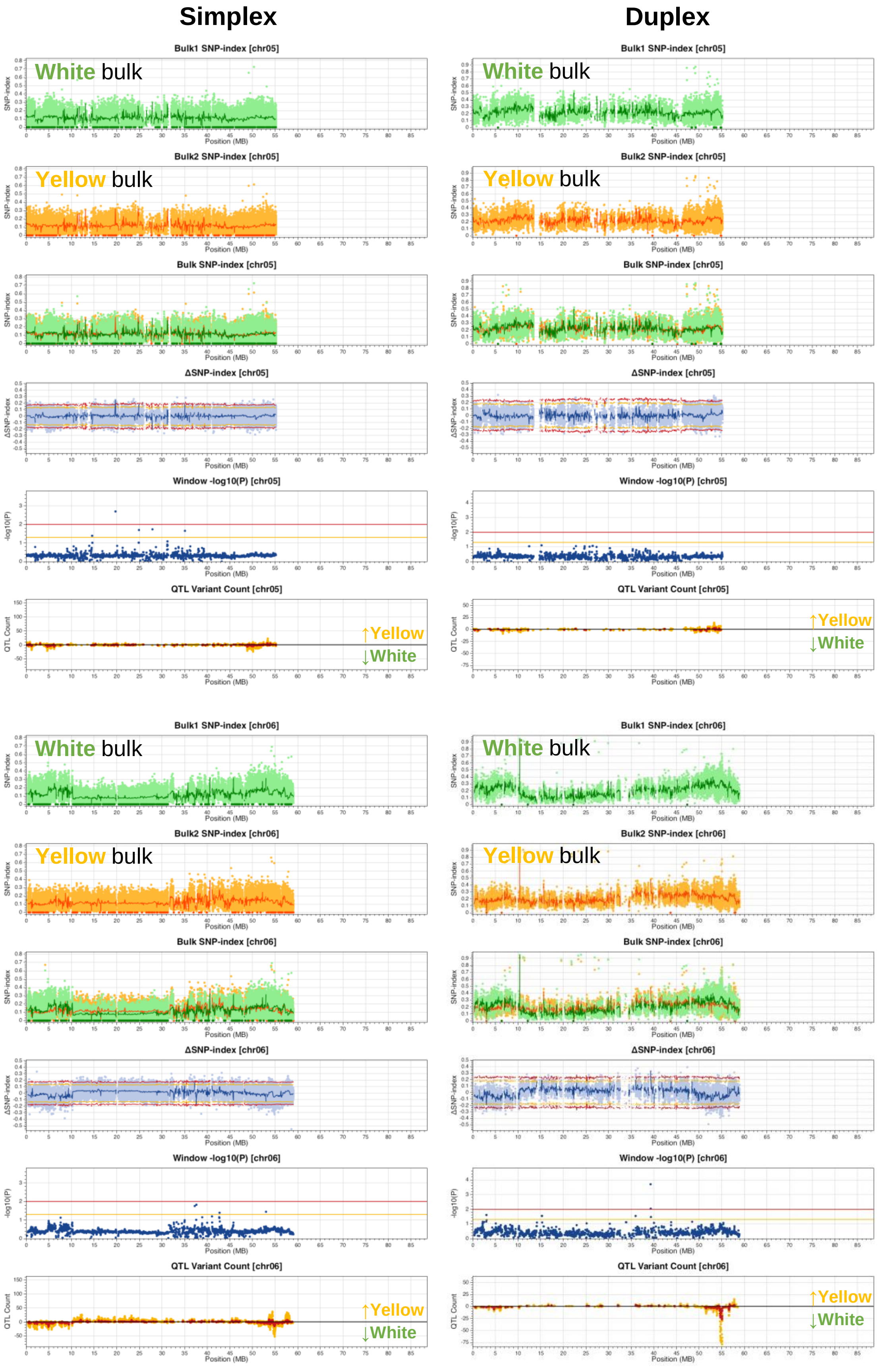

Supplemental Fig. 2. (continued)

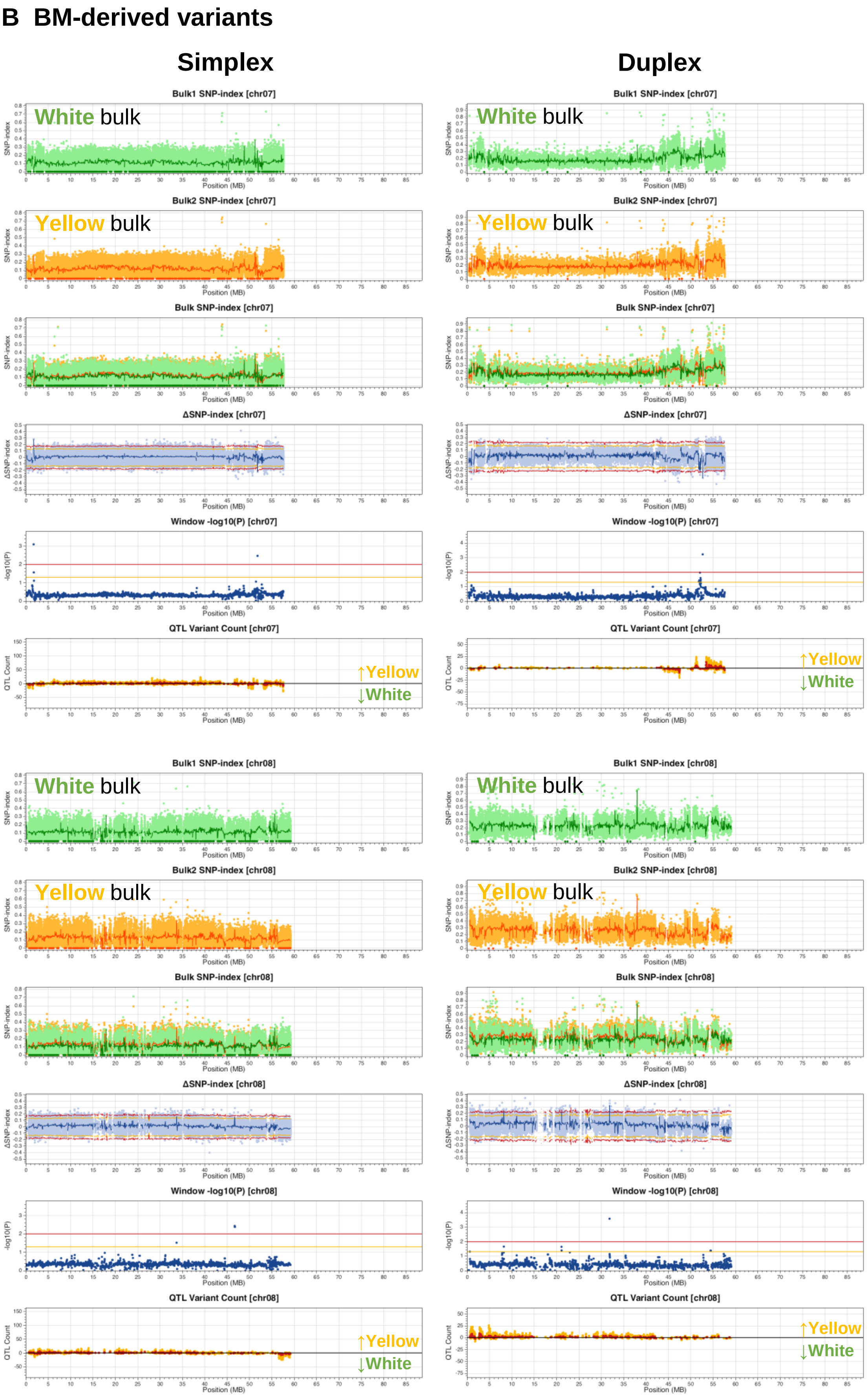

Supplemental Fig. 2. (continued)

B BM-derived variants

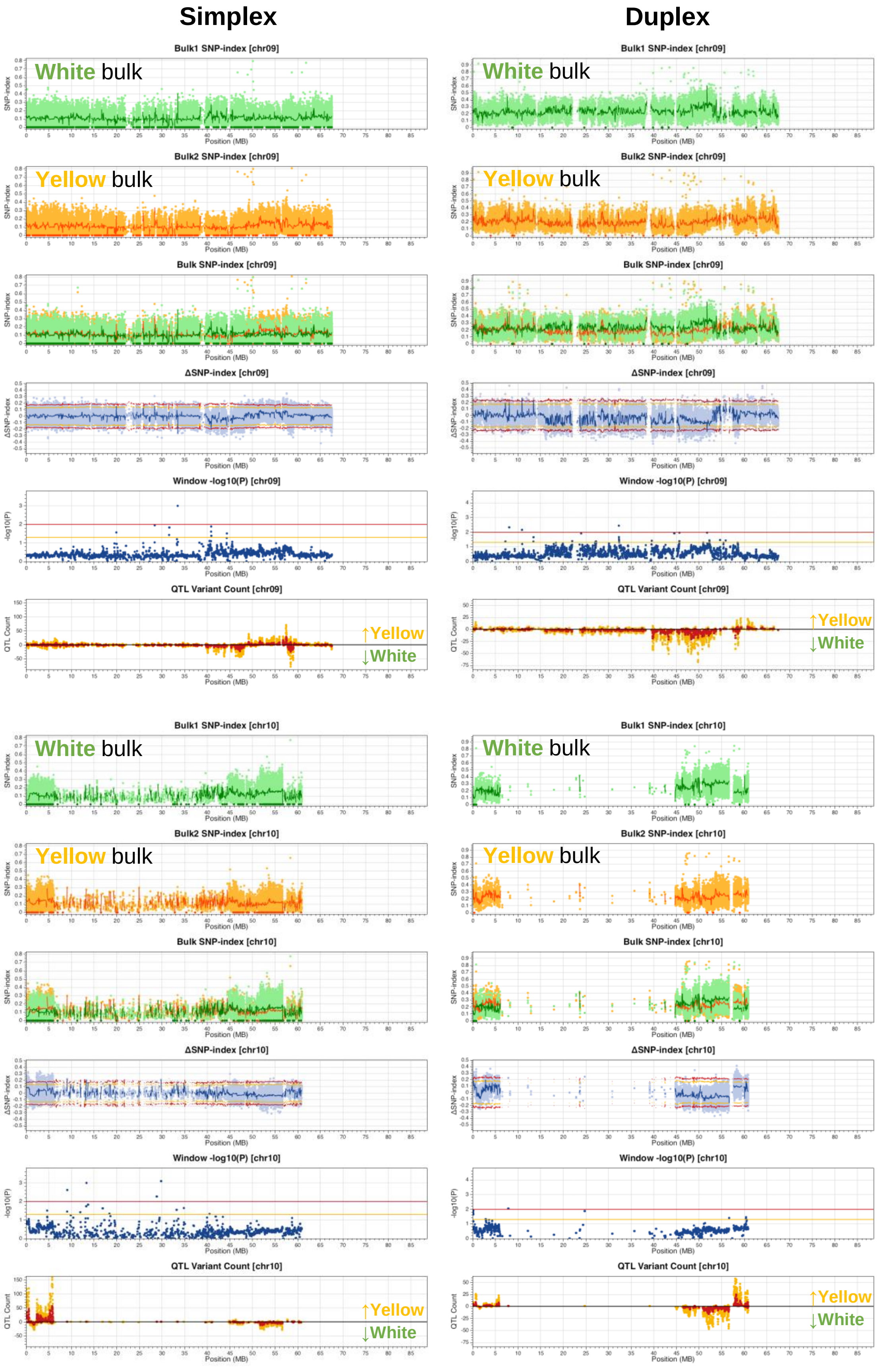

Supplemental Fig. 2. (continued)

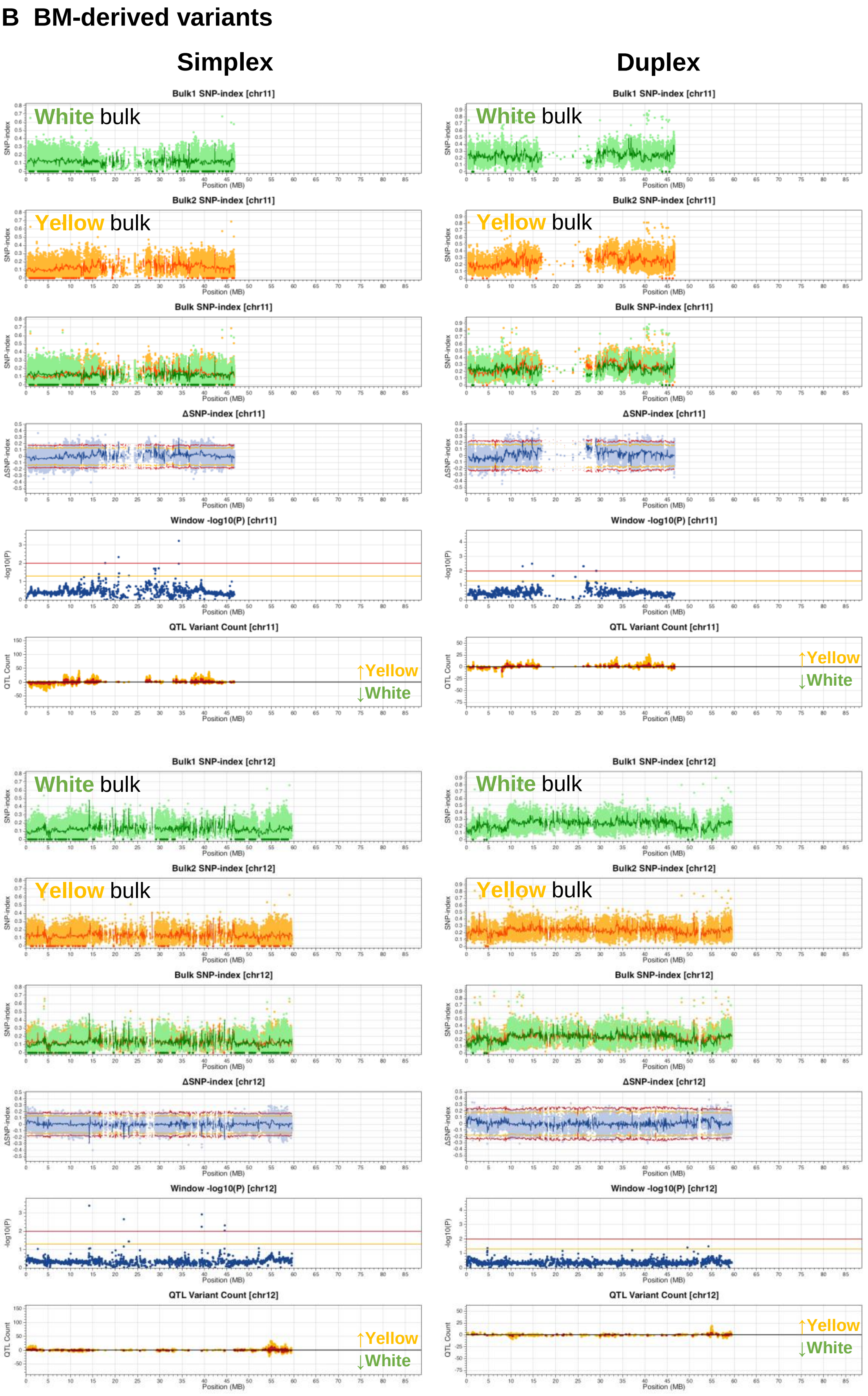

Supplemental Fig. 2. (continued)

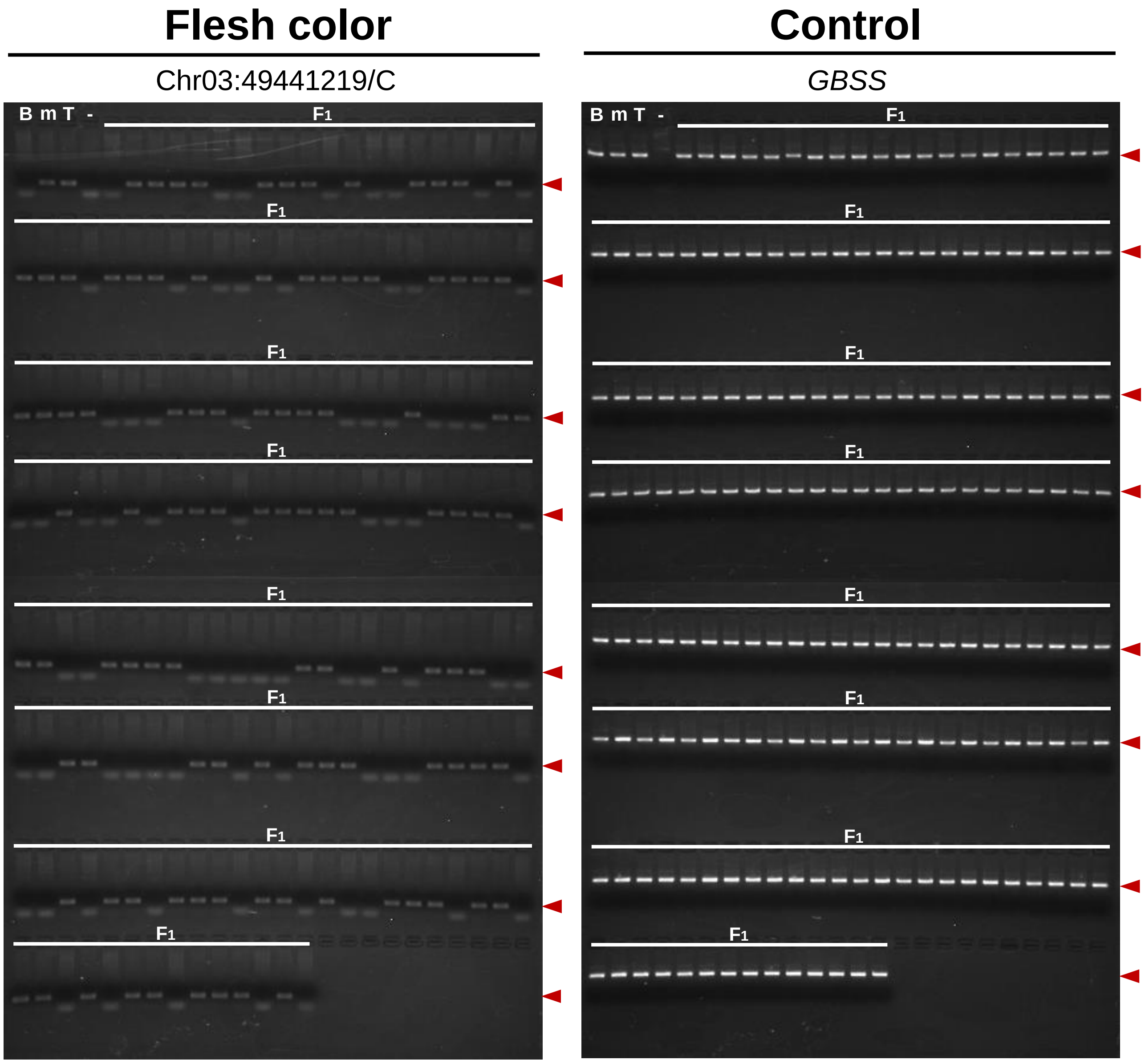

Supplemental Fig. 3. Agarose gel images of the DNA marker linked to yellow flesh color, Chr03:49441219/C, and the *GBSS* control for genotyping the F_1 progenies. Red arrowheads indicate the amplified fragments. B: BM, m: mixture of equal amounts of BM and TY DNA, T: TY, -: no template control, F_1: F_1 progenies.

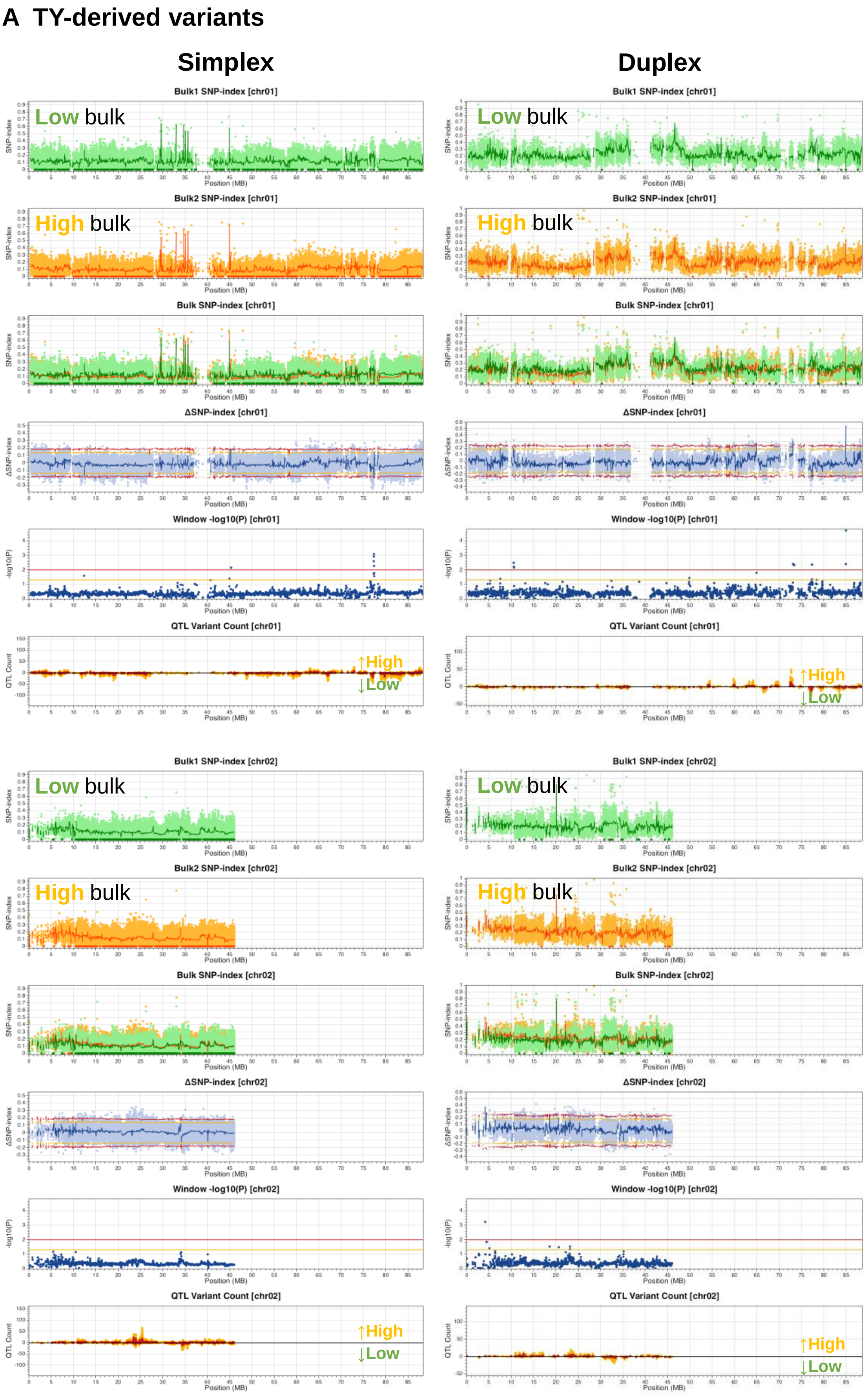

Supplemental Fig. 4. The genomic region regulating starch phosphorus content in F_1 progenies. (A) Polyploid QTL-seq analyses using each of TY-derived simplex and duplex variants. (B) Polyploid QTL-seq analyses using each of BM-derived simplex and duplex variants. The plots show SNP-indices of low bulk and high bulk, their superimposition, ΔSNP-index, window $-\log_{10}P$, and QTL variant count plot, as shown in Fig. 4. Red frames indicate a candidate region for QTL.

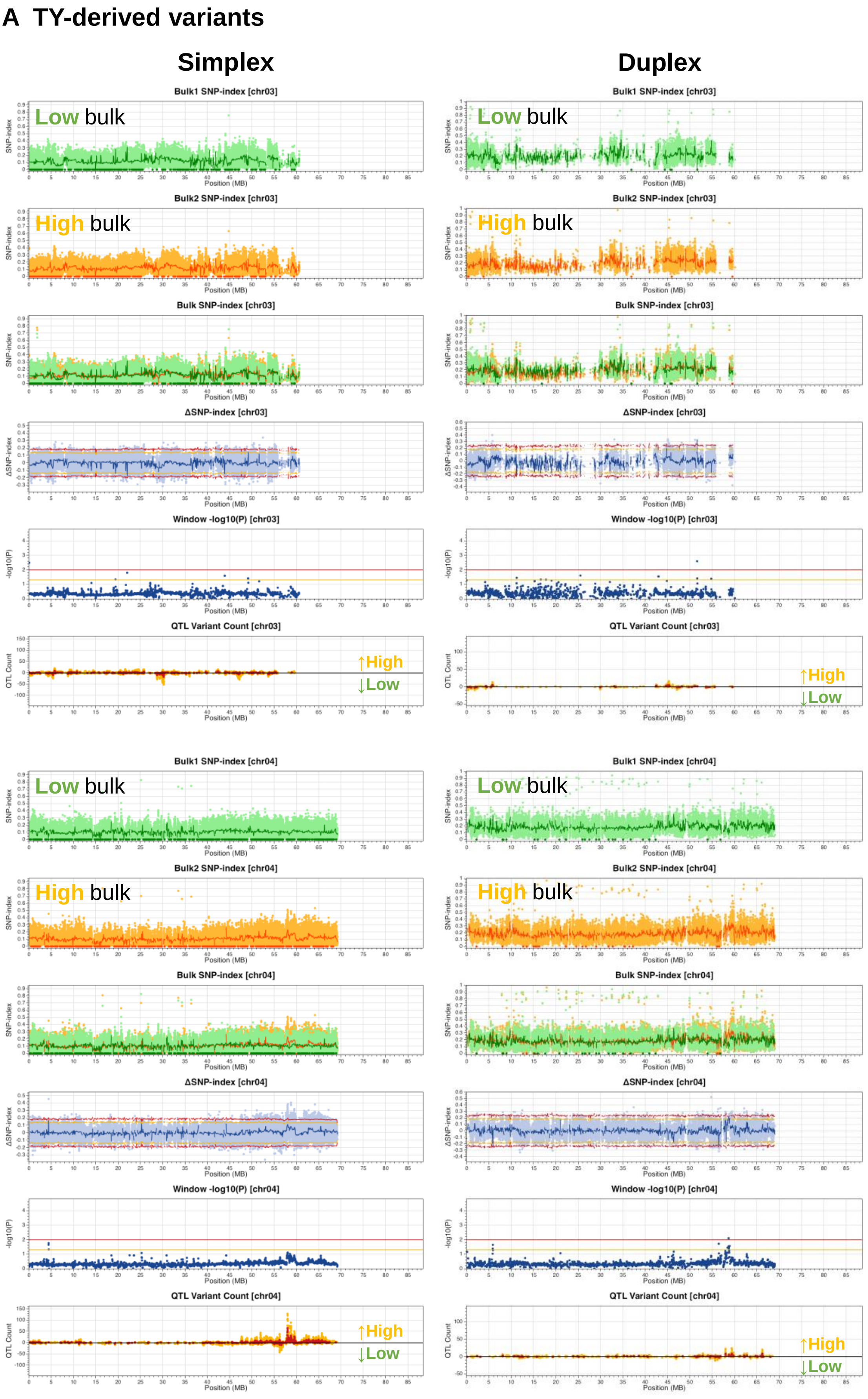

Supplemental Fig. 4. (continued)

A TY-derived variants

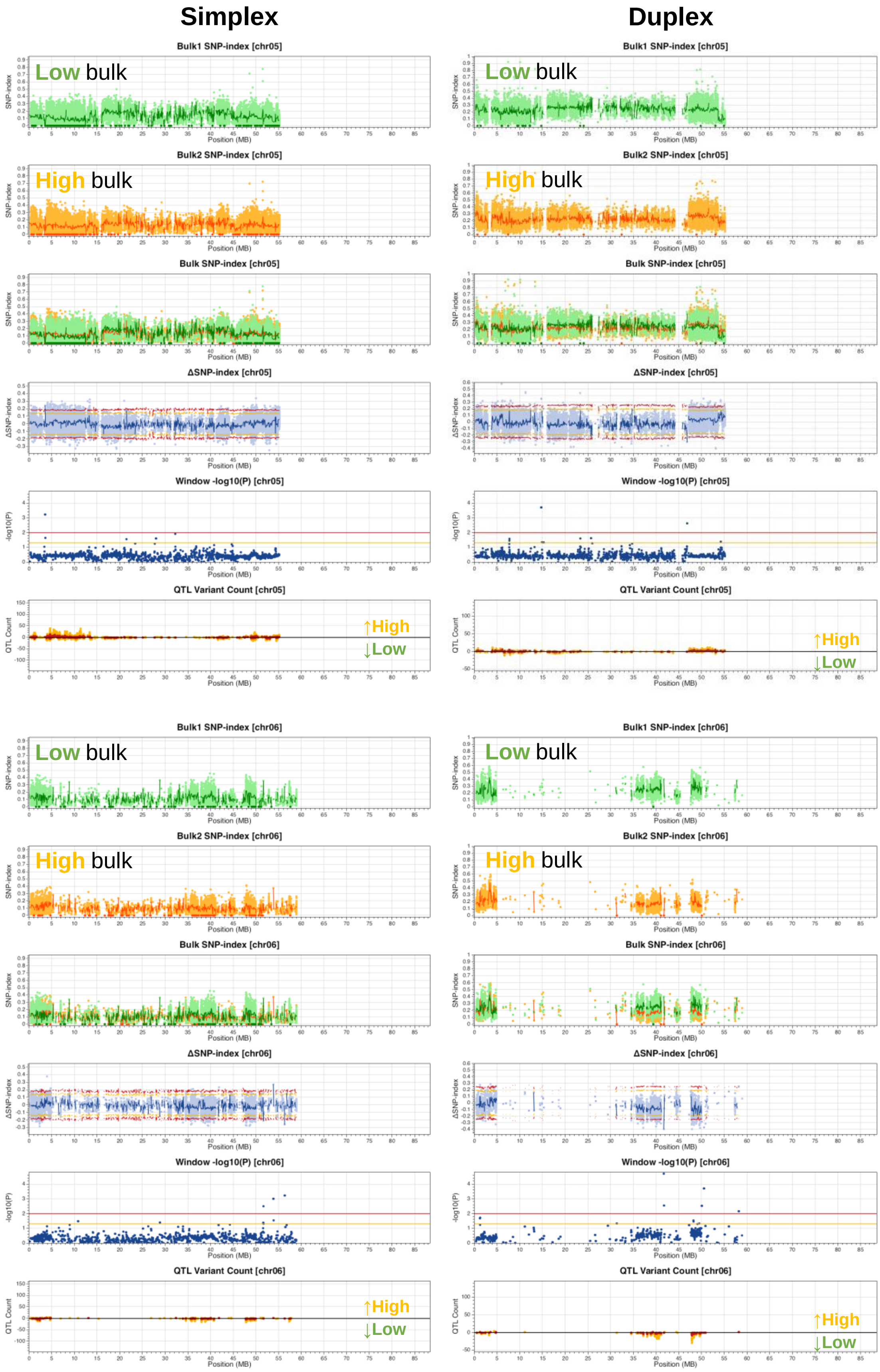

Supplemental Fig. 4. (continued)

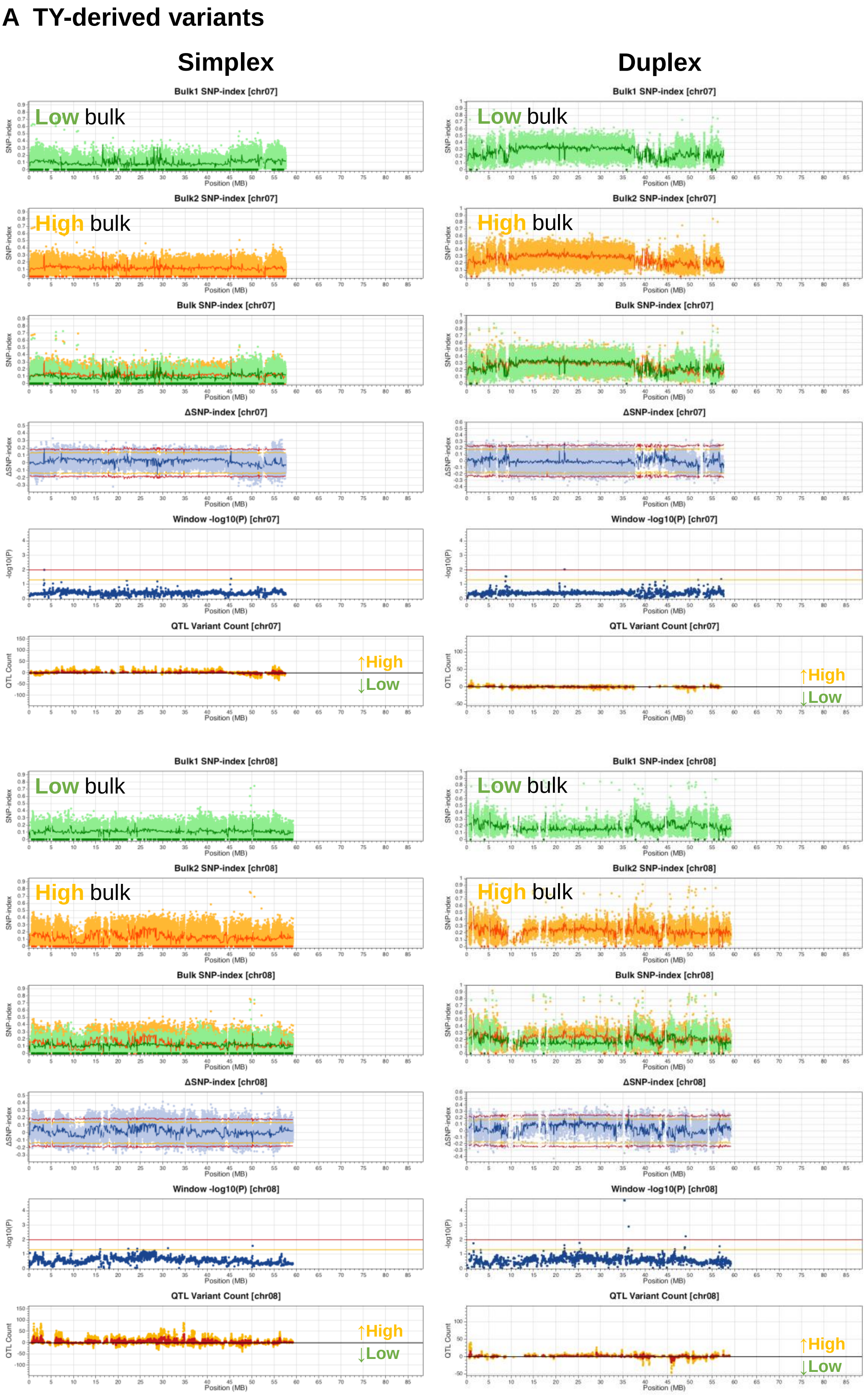

Supplemental Fig. 4. (continued)

A TY-derived variants

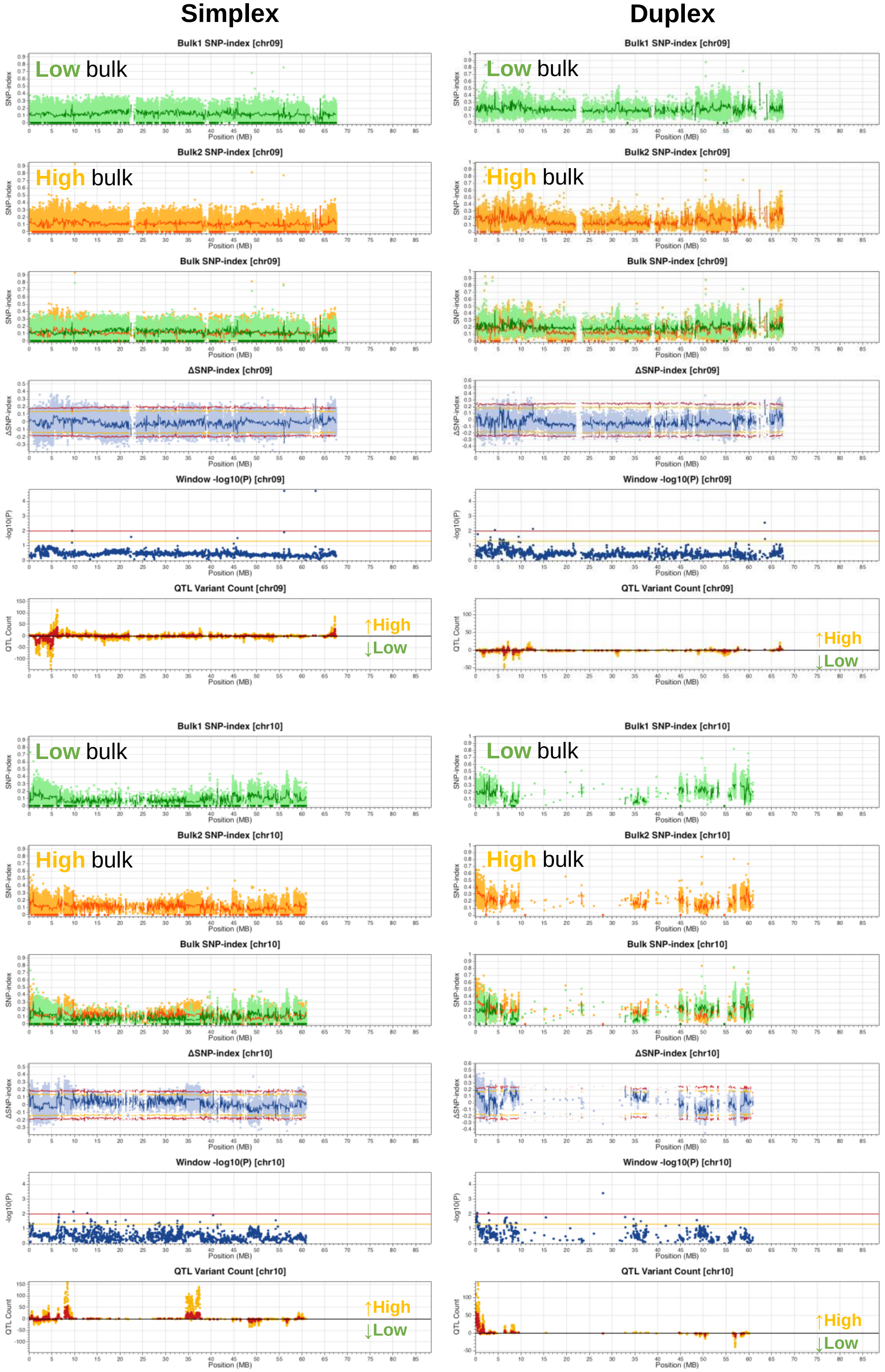

Supplemental Fig. 4. (continued)

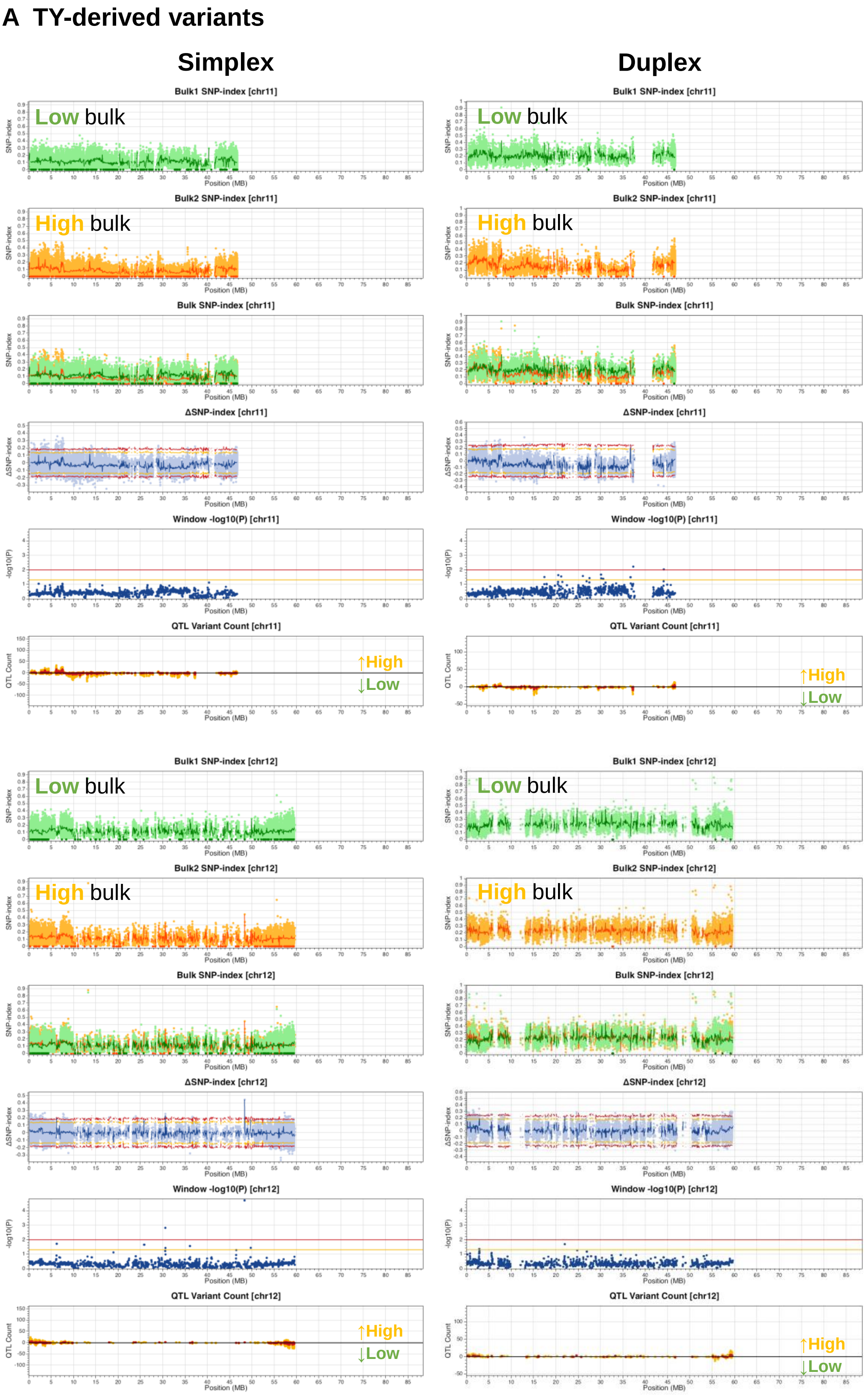

Supplemental Fig. 4. (continued)

B BM-derived variants

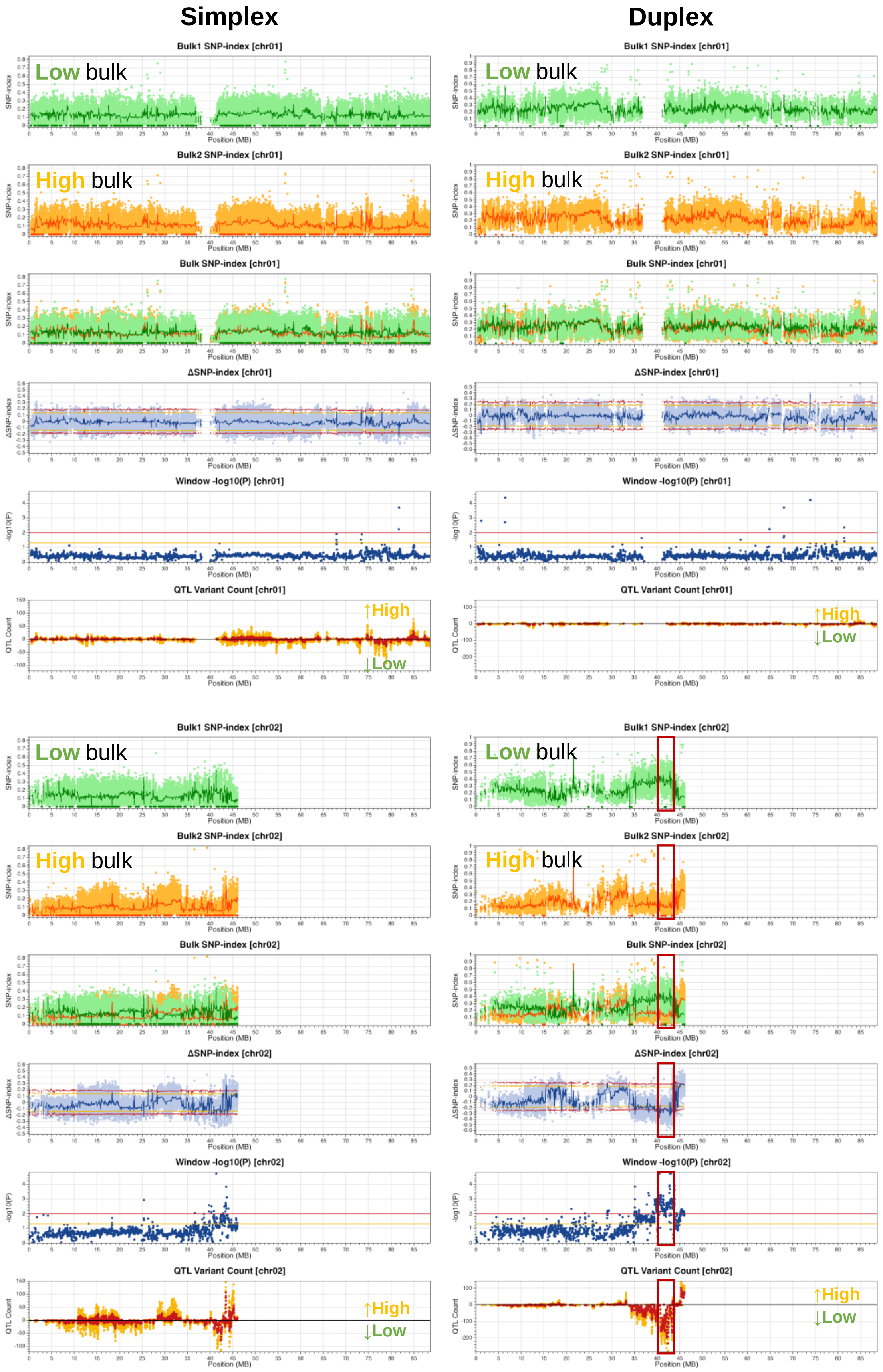

Supplemental Fig. 4. (continued)

B BM-derived variants

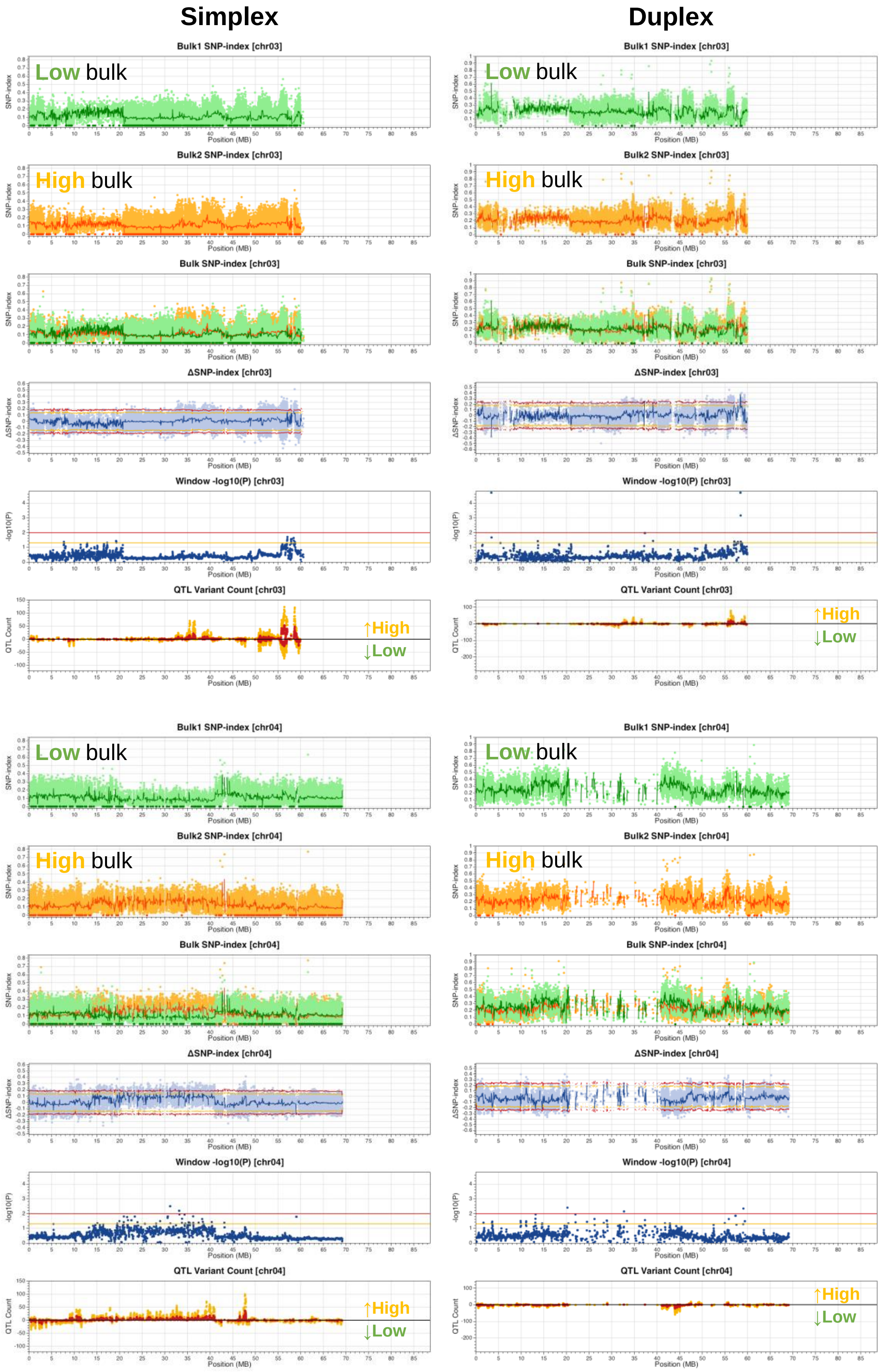

Supplemental Fig. 4. (continued)

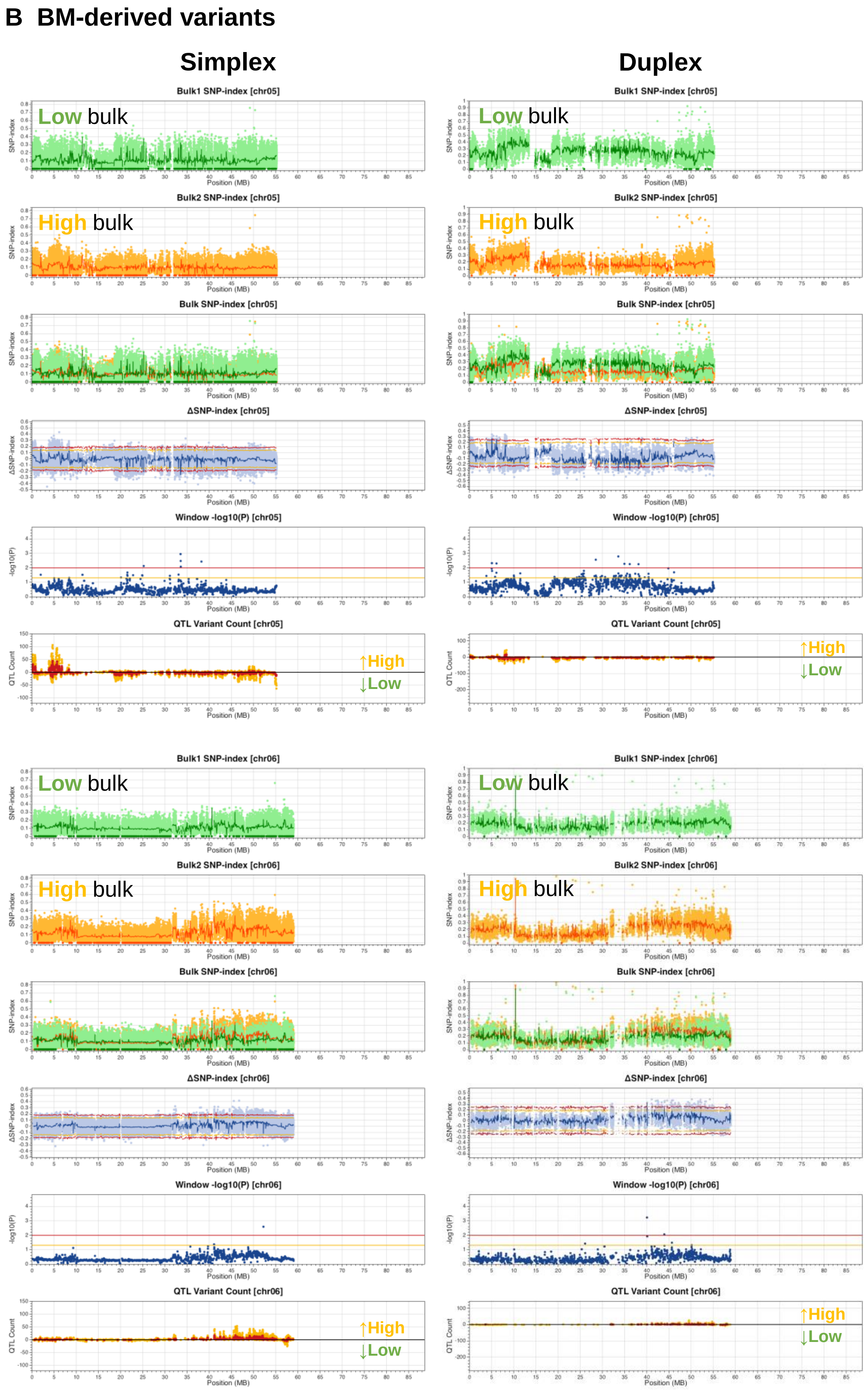

Supplemental Fig. 4. (continued)

B BM-derived variants

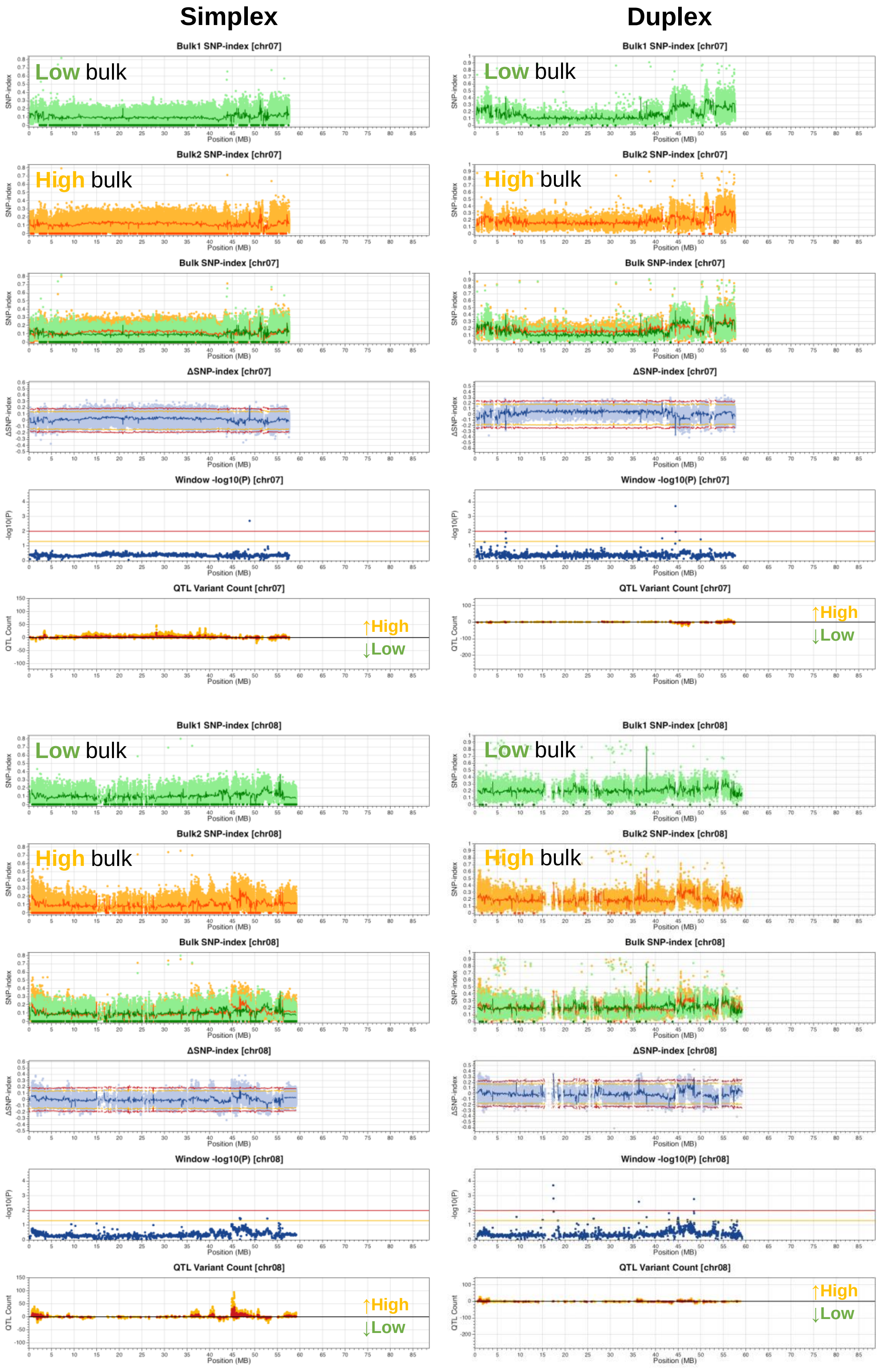

Supplemental Fig. 4. (continued)

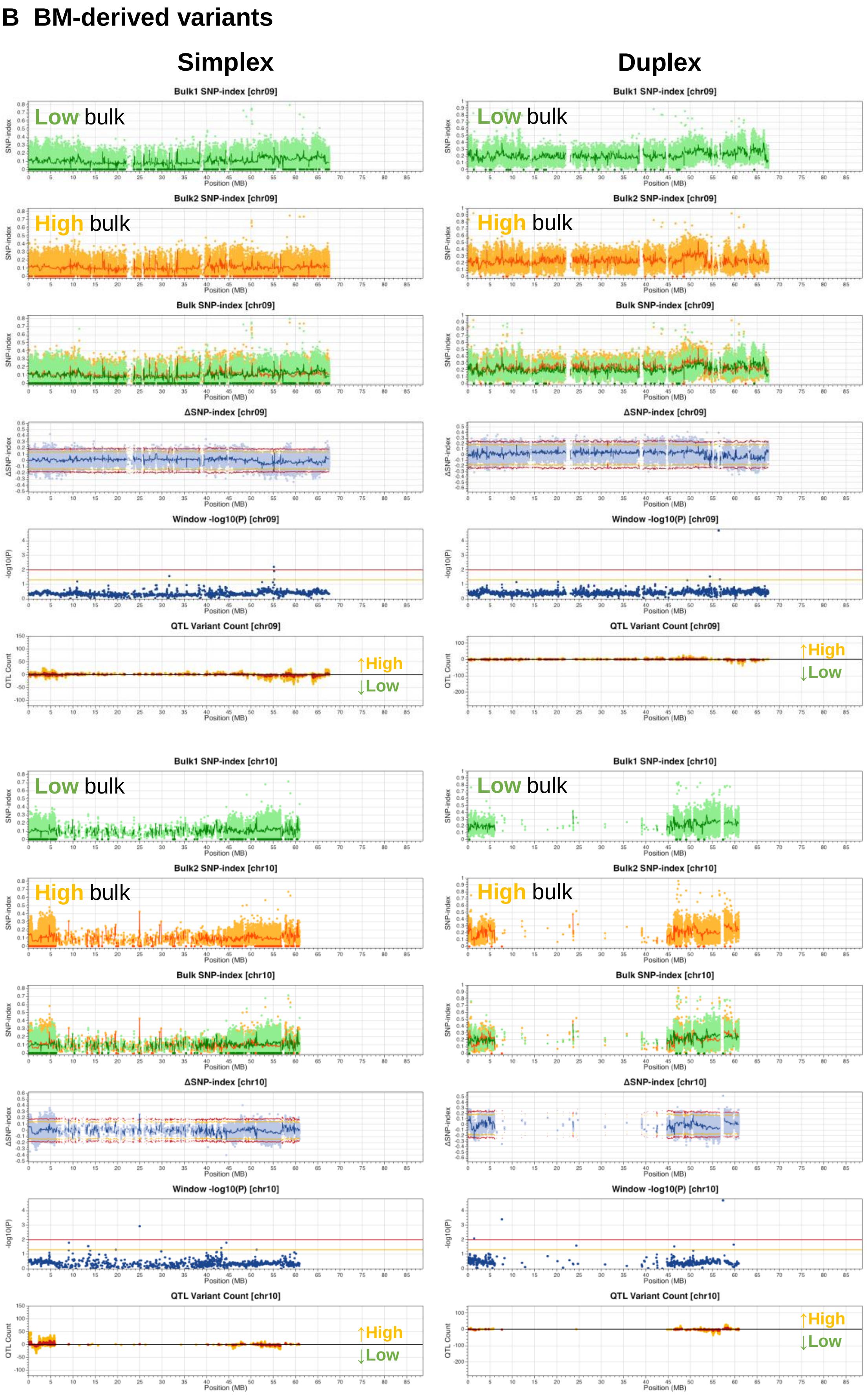

Supplemental Fig. 4. (continued)

B BM-derived variants

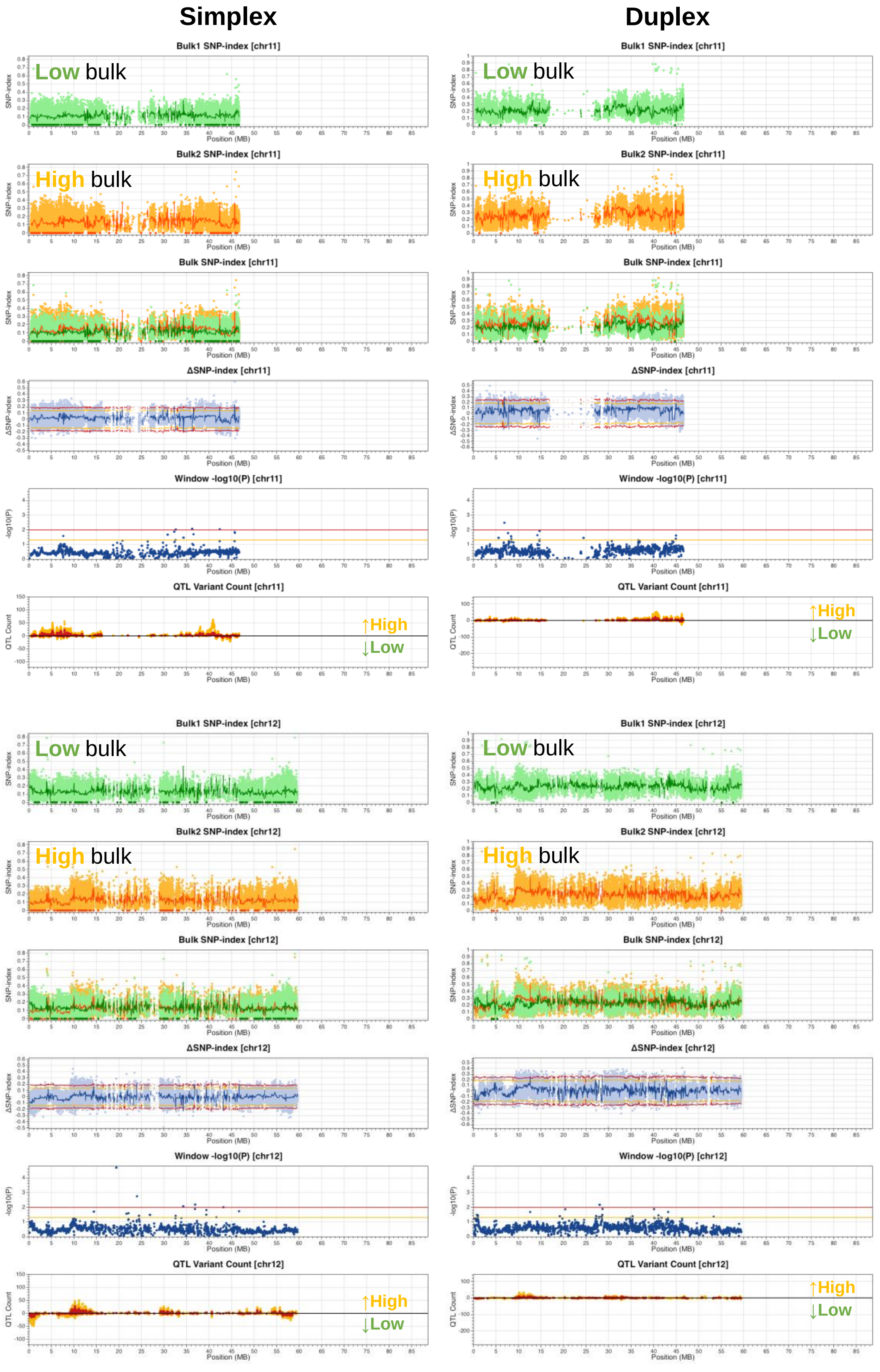

Supplemental Fig. 4. (continued)

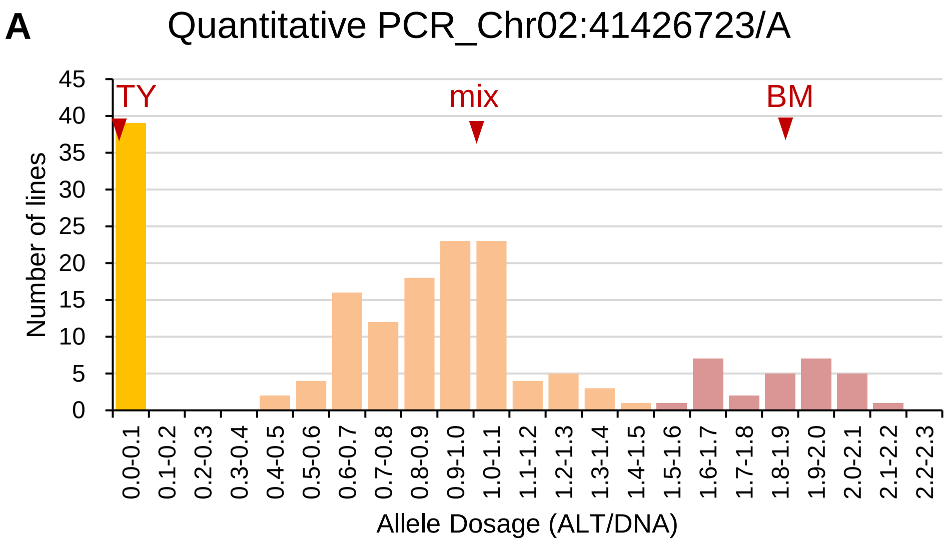

Supplemental Fig. 5. Dosage of the BM-derived, starch phosphorus content-associated allele determined by various genotyping methods. (A) Distribution of F_1 lines with specific alternative (ALT) allele dosage determined by the quantitative PCR with the Chr02:41426723/A marker. (B) Distribution of F_1 lines with specific peak height ratio of the ALT allele-derived fragment to the reference (REF) allele-derived fragment determined by the fragment analysis with the Chr03:41523385/A_ATL and Chr03:41523385/T_REF markers. Electropherograms of nulliplex (TY, REF:ALT allele dosage ratio 4:0), simplex (mixture, 3:1), and duplex (BM, 2:2) samples are shown. (C) Distribution of F_1 lines with specific read count ratio of the ALT base read to the total reads (REF + ALT) determined by amplicon sequence targeting three regions of Chr02:41322042, Chr02:41426723, and Chr02:41523385. Red arrowheads indicate the values of TY and BM, corresponding to nulliplex and duplex genotypes in terms of the BM-derived allele, respectively, as well as that of their equal amount mixture sample, whose allele dosage is equivalent to the simplex genotype. Orange, red, and purple denote the criteria for designating nulliplex, simplex, and duplex genotypes, respectively.

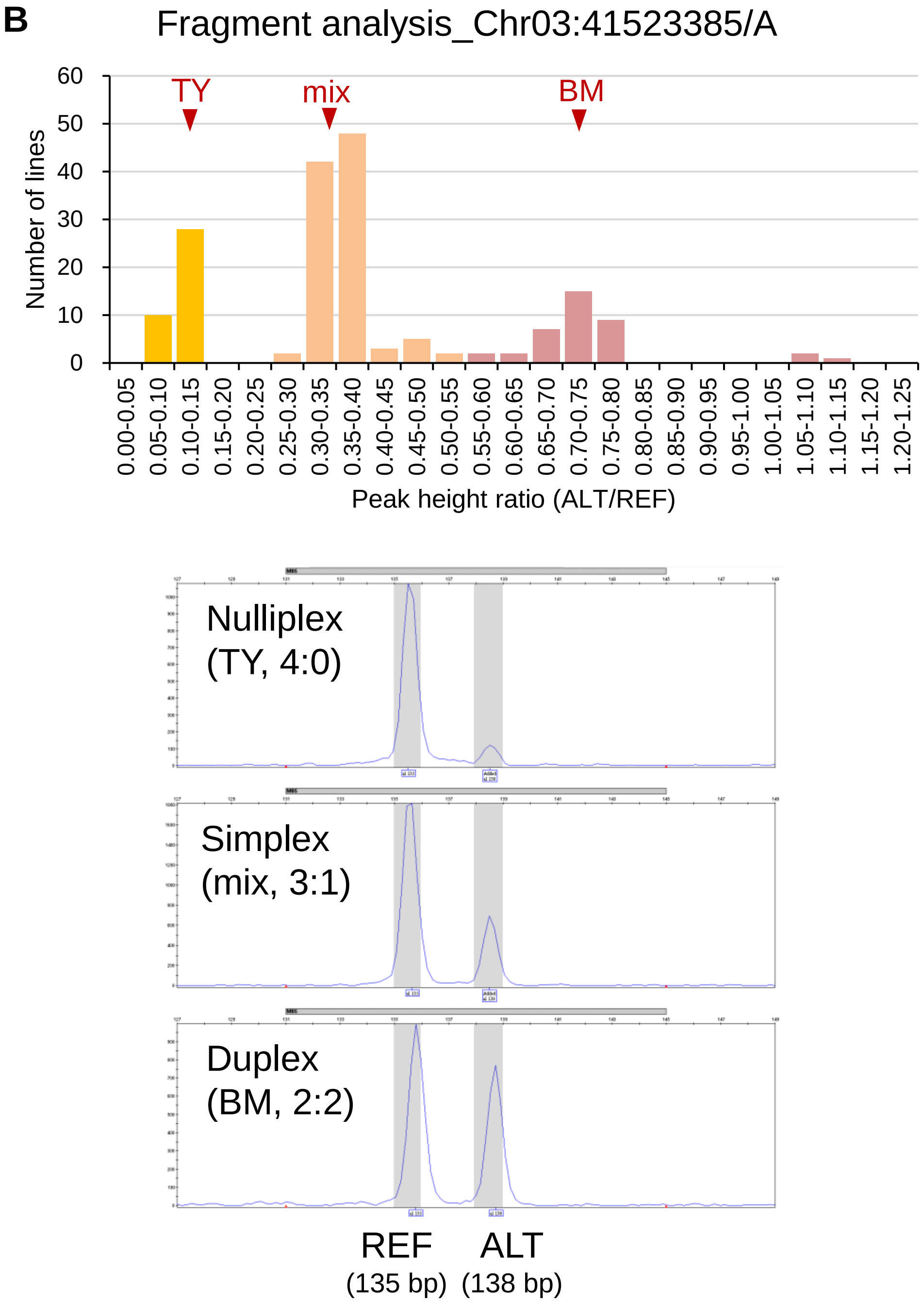

Supplemental Fig. 5. (continued)

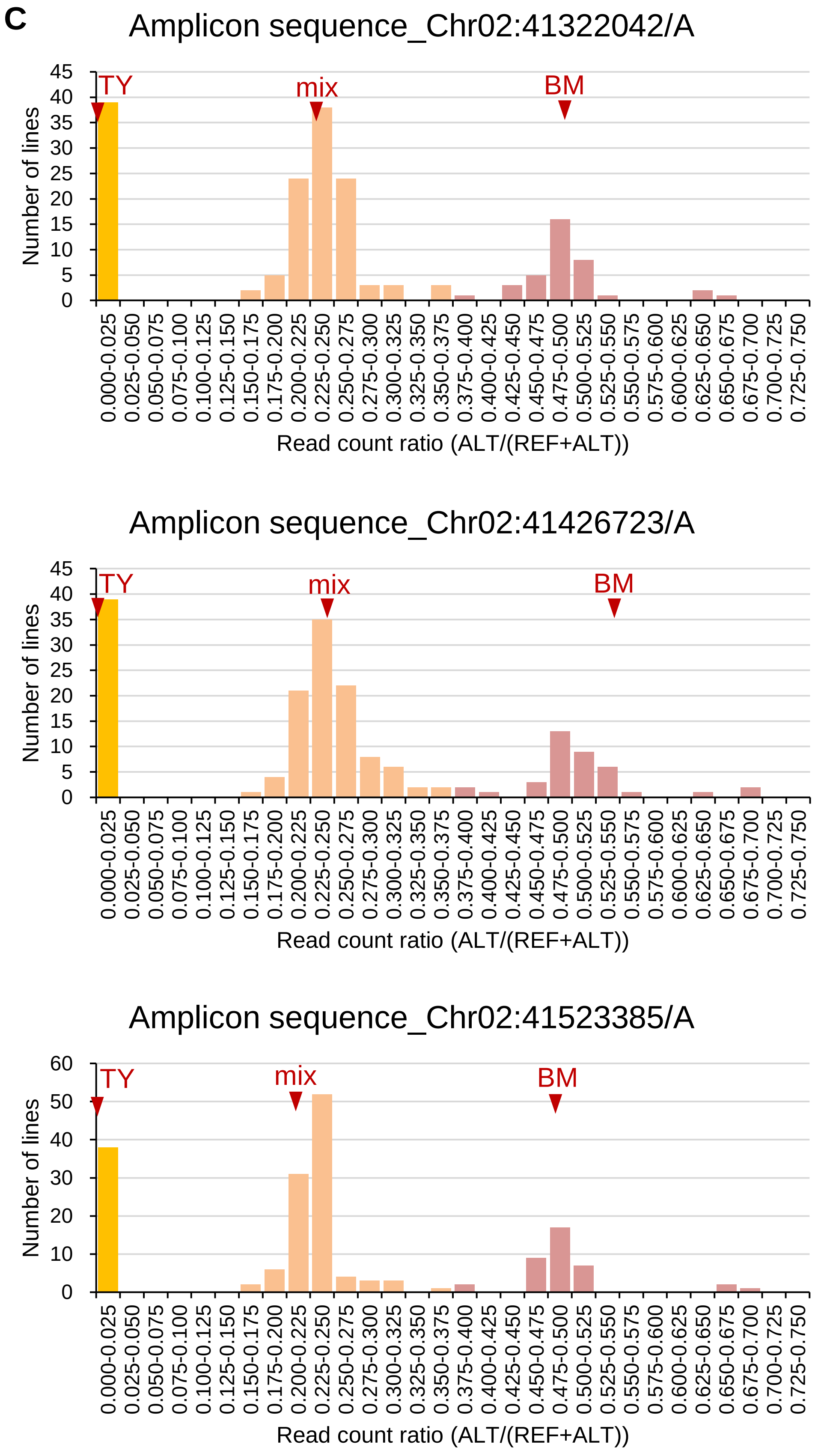

Supplemental Fig. 5. (continued)

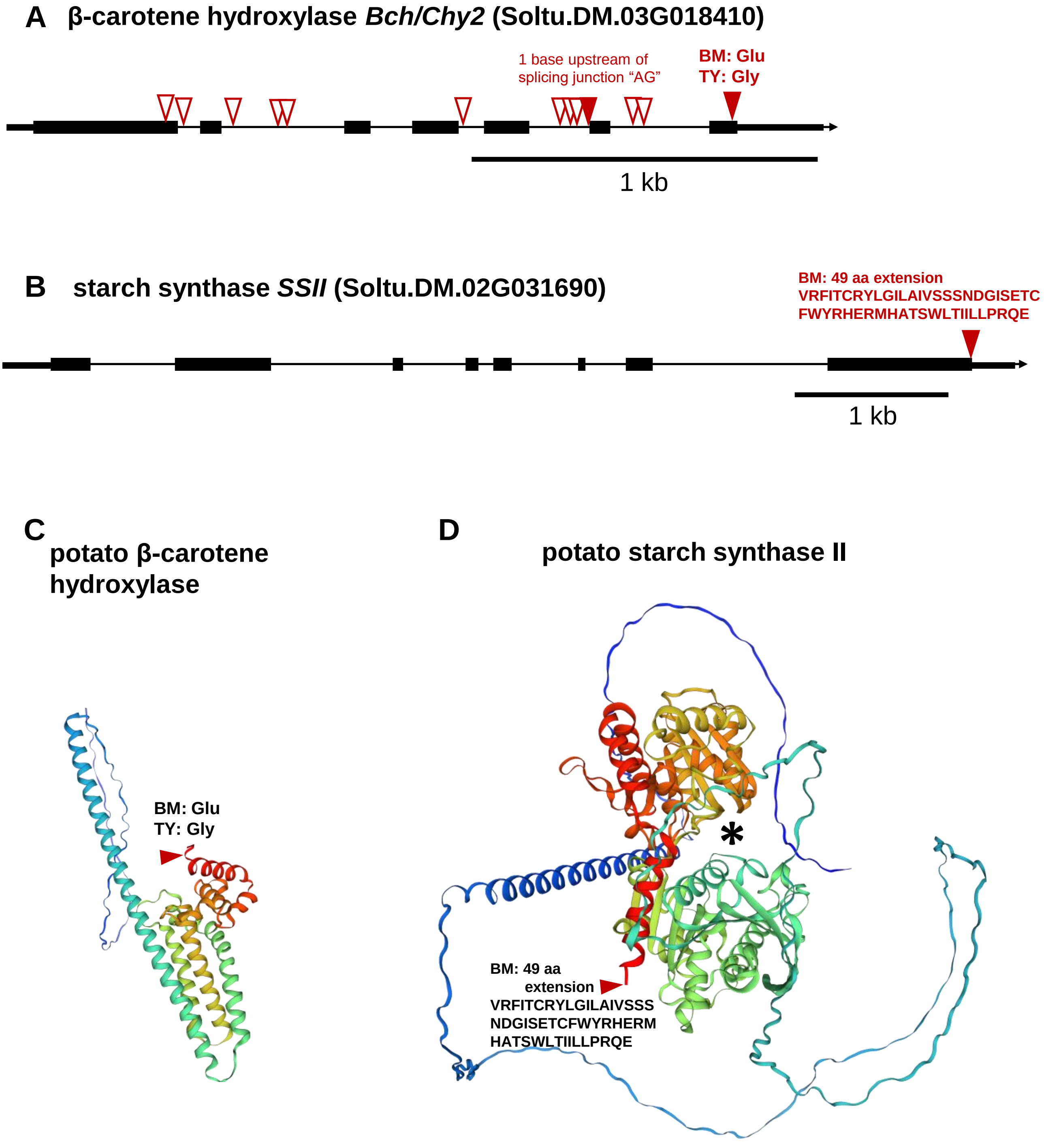

Supplemental Fig. 6. The location of the QTL-deduced variations. (A) The location of TY-derived polymorphisms in the *β-carotene hydroxylase* gene. (B) The location of a BM-derived polymorphism in the *starch synthase II* gene. Red and white arrowheads indicate polymorphisms leading to amino acid substitution or near the splicing junction and those without changes in amino acids or in the introns, respectively. (C) The structure of the potato β-carotene hydroxylase as estimated by SWISS-MODEL (SMTL id of D5M910.1.A). (D) The structure of the potato starch synthase II as estimated by SWISS-MODEL (SMTL id of Q43847.1.A). Red arrowheads indicate the site of the induced variations, and an asterisk denotes a catalytic groove.
